# IFN-γ signature enables selection of neoadjuvant treatment in patients with stage III melanoma

**DOI:** 10.1084/jem.20221952

**Published:** 2023-03-15

**Authors:** Irene L.M. Reijers, Disha Rao, Judith M. Versluis, Alexander M. Menzies, Petros Dimitriadis, Michel W. Wouters, Andrew J. Spillane, Willem M.C. Klop, Annegien Broeks, Linda J.W. Bosch, Marta Lopez-Yurda, Winan J. van Houdt, Robert V. Rawson, Lindsay G. Grijpink-Ongering, Maria Gonzalez, Sten Cornelissen, Jasper Bouwman, Joyce Sanders, Elsemieke Plasmeijer, Yannick S. Elshot, Richard A. Scolyer, Bart A. van de Wiel, Daniel S. Peeper, Alexander C.J. van Akkooi, Georgina V. Long, Christian U. Blank

**Affiliations:** 1 Department of Medical Oncology, Netherlands Cancer Institute, Amsterdam, Netherlands; 2 Molecular Oncology and Immunology, Netherlands Cancer Institute, Amsterdam, Netherlands; 3 Melanoma Institute Australia, The University of Sydney, Sydney, Australia; 4 Faculty of Medicine and Health, The University of Sydney, Sydney, Australia; 5 Department of Medical Oncology, Royal North Shore and Mater Hospitals, Sydney, Australia; 6 Department of Surgical Oncology, Netherlands Cancer Institute, Amsterdam, Netherlands; 7 Department of Biomedical Data Sciences, Leiden University Medical Center, Leiden, Netherlands; 8 Department of Breast and Melanoma Surgery, Royal North Shore and Mater Hospitals, Sydney, Australia; 9 Department of Head and Neck Surgical Oncology, Netherlands Cancer Institute, Amsterdam, Netherlands; 10 Core Facility and Molecular Pathology & Biobanking department, Netherlands Cancer Institute, Amsterdam, Netherlands; 11 Pathology and Molecular Diagnostics Department, Netherlands Cancer Institute, Amsterdam, Netherlands; 12 Biometrics Department, Netherlands Cancer Institute, Amsterdam, Netherlands; 13 Departments of Tissue Pathology and Diagnostic Oncology, Royal Prince Alfred Hospital and NSW Health Pathology, Sydney, Australia; 14 Department of Dermatology, Netherlands Cancer Institute, Amsterdam, Netherlands; 15 Department of Dermatology, Leiden University Medical Center, Leiden, Netherlands; 16 Department of Dermatology, Amsterdam UMC, University of Amsterdam, Amsterdam, Netherlands; 17 Charles Perkins Centre, The University of Sydney, Sydney, Australia; 18 Department of Pathology, Netherlands Cancer Institute, Amsterdam, Netherlands; 19 Department of Melanoma Surgical Oncology, Royal Prince Alfred Hospital, Sydney, Australia; 20 Department of internal medicine, Leiden University Medical Center, Leiden, Netherlands

## Abstract

Neoadjuvant ipilimumab + nivolumab has demonstrated high pathologic response rates in stage III melanoma. Patients with low intra-tumoral interferon-γ (IFN-γ) signatures are less likely to benefit. We show that domatinostat (a class I histone deacetylase inhibitor) addition to anti-PD-1 + anti-CTLA-4 increased the IFN-γ response and reduced tumor growth in our murine melanoma model, rationalizing evaluation in patients. To stratify patients into IFN-γ high and low cohorts, we developed a baseline IFN-γ signature expression algorithm, which was prospectively tested in the DONIMI trial. Patients with stage III melanoma and high intra-tumoral IFN-γ scores were randomized to neoadjuvant nivolumab or nivolumab + domatinostat, while patients with low IFN-γ scores received nivolumab + domatinostat or ipilimumab + nivolumab + domatinostat. Domatinostat addition to neoadjuvant nivolumab ± ipilimumab did not delay surgery but induced unexpected severe skin toxicity, hampering domatinostat dose escalation. At studied dose levels, domatinostat addition did not increase treatment efficacy. The baseline IFN-γ score adequately differentiated patients who were likely to benefit from nivolumab alone versus patients who require other therapies.

## Teaser

In the DONIMI trial, a baseline IFN-γ signature enables prospective selection of patients who can benefit from anti-PD-1 monotherapy. In contrast to results of our murine melanoma model, domatinostat (a class I HDAC inhibitor) does not add benefit to neoadjuvant anti-PD-1 ± anti-CTLA-4 in patients.

## Graphical Abstract

**Figure:**
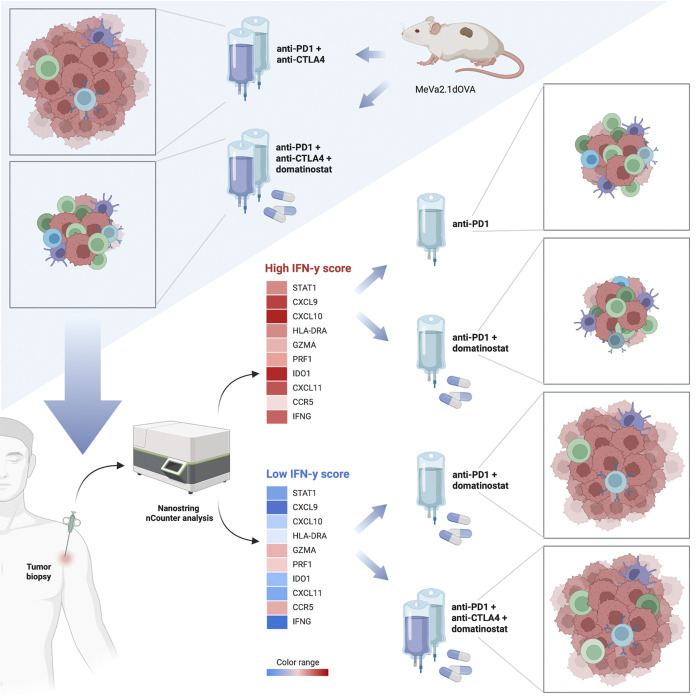

## Introduction

To date, standard treatment for patients with macroscopic stage III melanoma includes surgery followed by adjuvant immune checkpoint blockade (ICB) directed against programmed death-1 (anti-PD-1) or adjuvant BRAF/MEK-targeted therapy for those with BRAF-mutant melanoma. However, growing evidence from preclinical studies (Liu et al., 2016) and phase 1–2 clinical trials in stage III melanoma testing neoadjuvant ICB directed against PD-1 ± cytotoxic T lymphocyte antigen-4 (anti-CTLA-4; Blank et al., 2018; Patel et al., 2023) indicates that neoadjuvant ICB is superior to adjuvant ICB. Furthermore, several clinical trials have demonstrated impressive pathologic response rates that strongly correlated with relapse-free survival (RFS; Blank et al., 2018; Amaria et al., 2018; Rozeman et al., 2021; Blank et al., 2020; Menzies et al., 2021; Reijers et al., 2022b). A pooled analysis of the International Neoadjuvant Melanoma Consortium (INMC) demonstrated that relapses were rare in patients achieving a pathologic response (≤50% residual viable tumor) after neoadjuvant mono or combination ICB (2-yr RFS 94–100%), whereas patients without pathologic response were at higher risk of relapse (2-yr RFS 37%; Menzies et al., 2021). These data highlight the urgent need for identification of baseline biomarkers of response to identify patients who should be treated with mono versus combination ICB, or novel treatment combinations for patients deemed less likely to respond to the current most effective combination therapy in melanoma; ipilimumab (anti-CTLA-4) + nivolumab (anti-PD-1).

In our previous OpACIN-neo trial testing different dosing schedules of neoadjuvant ipilimumab + nivolumab in stage III melanoma, we identified the 10-gene IFN-γ signature (Ayers et al., 2017) as a predictive baseline biomarker for pathologic response (Rozeman et al., 2021). Therefore, and considering the risk of long-term immunotherapy-related adverse events after ICB therapy (e.g., endocrine toxicities and fatigue), we developed a baseline IFN-γ signature expression algorithm that differentiated patients who were more and less likely to respond to neoadjuvant ICB (Reijers et al., 2022a). We aimed to test treatment de-escalation in patients with a high IFN-γ signature gene expression score (IFN-γ score) in their baseline tumor biopsy, and treatment escalation in patients with a low IFN-γ score (e.g., administering alternative double or triple treatment combinations).

Histone deacetylase inhibitors (HDACi) are epigenetic modifiers known to have direct anti-tumor effects in melanoma (Yeon et al., 2020). In addition, the immune-modulatory properties of HDACi make them potential candidates to test in combination with ICB (Vo et al., 2009; McCaw et al., 2017). Despite encouraging preclinical evidence, pan-HDAC inhibition has been met with limited success in the clinic owing to increased toxicities and detrimental effects on immune cell populations (Haas et al., 2014; Wong et al., 2014; Ibrahim et al., 2016; Chang et al., 2021). This could be overcome by the use of inhibitors that selectively target class I HDACs, since the inhibition of class I HDACs has been shown to increase the expression of predicted tumor neoantigens and MHC I, and inhibit the frequency of suppressive immune cells (Shen et al., 2012; Deng et al., 2019; Bretz et al., 2019; Truong et al., 2021).

Domatinostat, a selective class I HDAC inhibitor, has been shown to synergize with PD-L1 blockade in murine colon carcinoma models (Bretz et al., 2019). Importantly, treatment with domatinostat has been shown to increase IFN-γ response scores in preclinical non-melanoma models and in patients with advanced cutaneous melanoma (Bretz et al., 2019). We therefore postulated that domatinostat might synergize with anti-PD-1 ± anti-CTLA-4 in patients with stage III melanoma and a low IFN-γ score in their baseline tumor biopsy. However, there is limited preclinical evidence for the combination of domatinostat with PD-1 and CTLA-4 blockade.

Here, we present preclinical evidence for combining domatinostat with anti-PD-1 ± anti-CTLA-4 in a melanoma tumor model, and report the first results of the DONIMI trial (NCT04133948). This phase 1b trial investigated the feasibility, safety, and efficacy of different combinations of neoadjuvant nivolumab ± ipilimumab with domatinostat in patients with stage III melanoma, stratified according to the IFN-γ score from a baseline tumor biopsy.

## Results

### Domatinostat addition to checkpoint blockade controls growth of murine melanoma tumors through immune modulation

**Figure 1.**
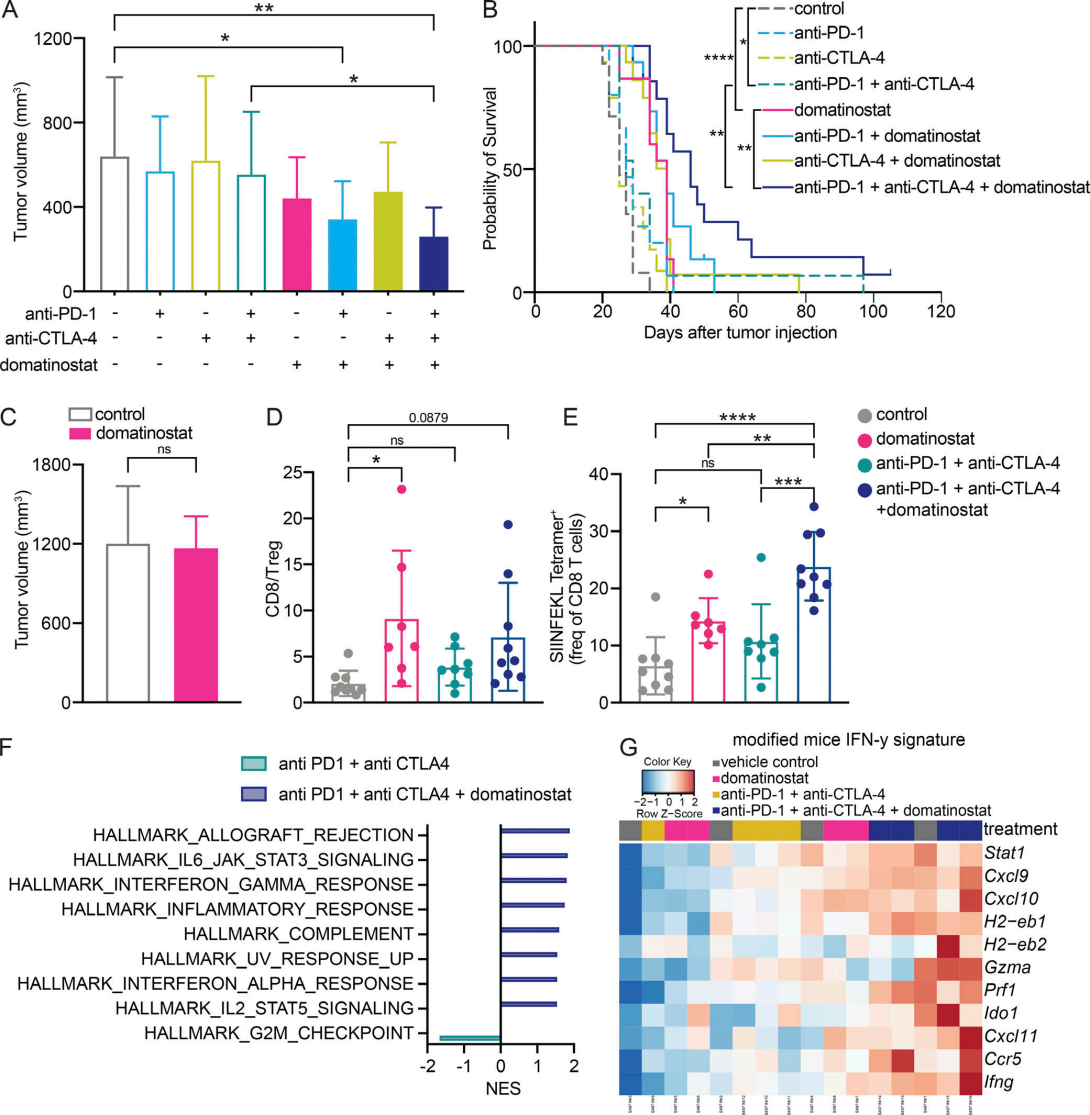
**Domatinostat addition to checkpoint blockade controls growth of murine melanoma tumors through immune-modulation. (A and B)** Tumor volume across groups at endpoint (day 20 after tumor injection; data represents average of *n* = 15 mice per group; exception: vehicle control and triple combo treatment with *n* = 14 mice per group; A) and survival of MeVa2.1.dOVA tumor bearing immune-competent C57BL/6 mice treated with combinations of domatinostat, anti-PD-1, and anti-CTLA-4 or vehicle control (B). Truncated events are early termination due to ulceration or due to complete response (*n* = 15 mice per group; exception: vehicle control and triple combo treatment with *n* = 14 mice per group). Survival analysis was performed using a log-rank test. **(C)** Tumor volume at endpoint (day 32 after tumor injection) of MeVa2.1.dOVA tumor bearing immune-compromised NSG mice treated with domatinostat or vehicle control (data represents average of *n* = 13 mice per group). Groups were compared using two-tailed unpaired Student’s *t* test. **(D and E)** Ratio of CD8^+^ T cells and FoxP3^+^CD4^+^ regulatory T cells (Tregs; *n* = 7–9 mice per group; D), frequency of tumor-reactive SIINFEKL tetramer^+^ cells in the tumor after 13 d of treatment with domatinostat ± anti-PD-1 + anti-CTLA-4, as determined by flow cytometry analysis (*n* = 7–9 mice per group; each dot represents tumor from one mouse; E). **(F)** Gene set enrichment analysis depicting significantly enriched (at FDR < 25%) Hallmark gene sets measured using RNA sequencing in tumors harvested after 13 d of treatment with domatinostat ± anti-PD-1 + anti-CTLA-4 (*n* = 4 mice per group). **(G)** Heatmap depicting normalized z-score (obtained from RNA sequencing analysis) of expression profiles of genes associated with IFN-γ response (modified to replace human genes with mice orthologs) in MeVa2.1.dOVA tumors after 13 d of treatment with domatinostat ± anti-PD-1 + anti-CTLA-4 (*n* = 4 mice per group). Error bars represent SD. Unless otherwise stated, statistical significance was estimated using one-way ANOVA followed by Sidak’s multiple comparisons test. ns, not significant; *, P value < 0.05; **, P value < 0.01; ***, P value < 0.001; ****, P value < 0.0001.

**Figure S1.**
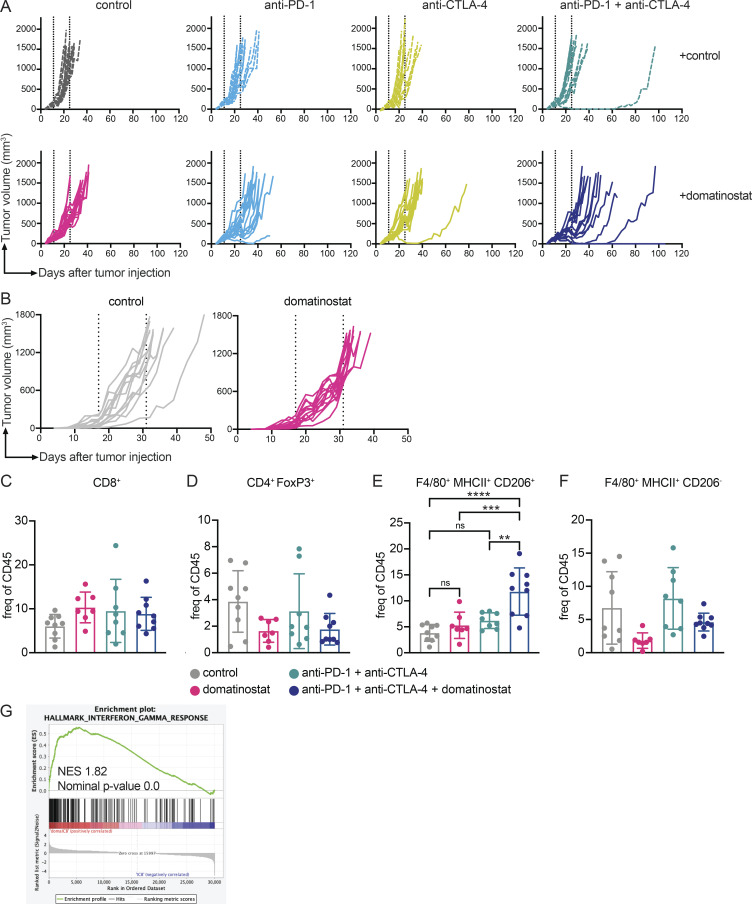
**Domatinostat addition to checkpoint blockade controls growth of murine melanoma tumors through immune modulation.** **(A)** MeVa2.1.dOVA tumor volume across days of individual C57BL/6 mice treated with combinations of domatinostat, anti-PD-1, and anti-CTLA-4 or vehicle control (*n* = 15 mice per group; exception: vehicle control and triple combo treatment with *n* = 14 mice per group). **(B)** MeVa2.1.dOVA tumor volume across days of individual NSG mice treated with domatinostat or vehicle control (*n* = 13 mice per group). Dotted line indicates treatment duration. **(C–F)** Frequency of (C) CD8^+^ T cells, (D) CD4^+^ FoxP3^+^ Tregs, (E) CD206^+^, and (F) CD206^–^ macrophages in the tumor, harvested after 13 d of treatment with domatinostat ± anti-PD-1 + anti-CTLA-4, as determined by flow cytometry analysis (*n* = 7–9 mice per group). Each dot represents tumor from one mouse. **(G)** Gene set enrichment analysis depicting Hallmark IFN-γ response, obtained using RNA sequencing analysis, in MeVa2.1.dOVA tumors after 13 d of treatment with domatinostat ± anti-PD-1 + anti-CTLA-4 (*n* = 4 mice per group). Statistical significance was estimated using one-way ANOVA followed by Sidak’s multiple comparisons test. Error bars represent SD. ns, not significant; **, P value < 0.01; ***, P value < 0.001; ****, P value < 0.0001.

It has been shown that domatinostat, in combination with PD-L1 blockade, results in tumor growth control in murine models of colon carcinoma (Bretz et al., 2019). However, its efficacy, especially in combination with anti-PD-1 ± anti-CTLA-4, in murine melanoma is not known. We therefore evaluated potential combination approaches, using domatinostat in the BRAF^V600E^ PTEN^−/−^-driven MeVa2.1.dOVA murine melanoma model, which was developed by our group (Rao et al., 2023). Addition of domatinostat to either anti-PD-1 alone or anti-PD-1 + anti-CTLA-4 significantly lowered tumor volume compared with vehicle control (Fig. 1 A and Fig. S1 A). Moreover, combination of domatinostat and anti-PD-1 + anti-CTLA-4 resulted in significantly prolonged survival of mice compared with either treatment alone (Fig. 1 B).

Since treatment with domatinostat alone was sufficient to observe a trend for slow tumor growth (Fig. 1 A and Fig. S1 A) and a significantly prolonged survival (Fig. 1 B) of MeVa2.1.dOVA-bearing mice, we assessed whether the efficacy of domatinostat could be attributed to direct tumor cell killing or immunomodulation. For this, we treated tumor-bearing immune-deficient NOD-scid IL2rγ^null^ (NSG) mice with domatinostat. There was no effect on tumor volume, indicating that tumor growth control by domatinostat requires the presence of an intact immune system and is not mediated via direct tumor cell killing (Fig. 1 C and Fig. S1 B).

Flow cytometry analysis showed an increased ratio of CD8^+^ T cells to FoxP3^+^ regulatory T cells (Tregs) in the tumor upon treatment with domatinostat (Fig. 1 D). Such an increased ratio could evolve from increased CD8^+^ T cell tumor infiltration or reduced presence of FoxP3^+^ Tregs. However, evaluating the sub-populations, we did not observe significant differences in the frequency of CD8^+^ T cells or CD4^+^ FoxP3^+^ Tregs in the tumor (Fig. S1, C and D). Interestingly, we observed a significant increase in the frequency of H-2Kb SIINFEKL tetramer positive CD8^+^ T cells upon domatinostat, and to a larger extent in domatinostat + anti-PD-1 + anti-CTLA-4–treated mice (Fig. 1 E), indicating an increase in tumor-reactive T cells upon treatment. In the analysis of the myeloid compartment, there was a significant increase in the frequency of CD206^+^ macrophages, and no significant change in the frequency of CD206^−^ macrophages in the tumor of mice treated with the triple combination therapy (Fig. S1, E and F), indicating a shift toward M2 macrophages.

To obtain additional insights into the immune-modulatory properties of domatinostat, we evaluated mRNA expression profiles of MeVa2.1.dOVA tumors treated with combination of anti-PD-1 + anti-CTLA-4 and domatinostat, using RNA sequencing. There was a significant enrichment of gene sets associated with pro-inflammatory response upon the addition of domatinostat to dual ICB (Fig. 1 F), and an increase in the expression of genes associated with IFN-γ response (Fig. 1 G and Fig. S1 G).

Twice daily treatment with domatinostat for 2 wk did not cause any body weight loss (data not shown), or behavioral changes such as shivering, pale skin, or excessive grooming in both immune-competent and immune-compromised mice, indicating no toxicity. We therefore concluded that inhibition of class I–specific HDAC using domatinostat controls tumor growth owing to enhanced anti-tumor immune responses, while mitigating the toxicities seen using pan-HDAC inhibition (Wong et al., 2014). In summary, analyses in our murine melanoma model were in line with other preclinical data (Bretz et al., 2019). The data from our preclinical melanoma model, along with the existing results in other preclinical models and clinical data indicating increased expression of the IFN-γ score in patients with advanced cutaneous melanoma (Bretz et al., 2019), together provided a strong rationale to test the combination of domatinostat with ICB in patients with melanoma.

### The DONIMI trial

#### Baseline characteristics

**Figure 2.**
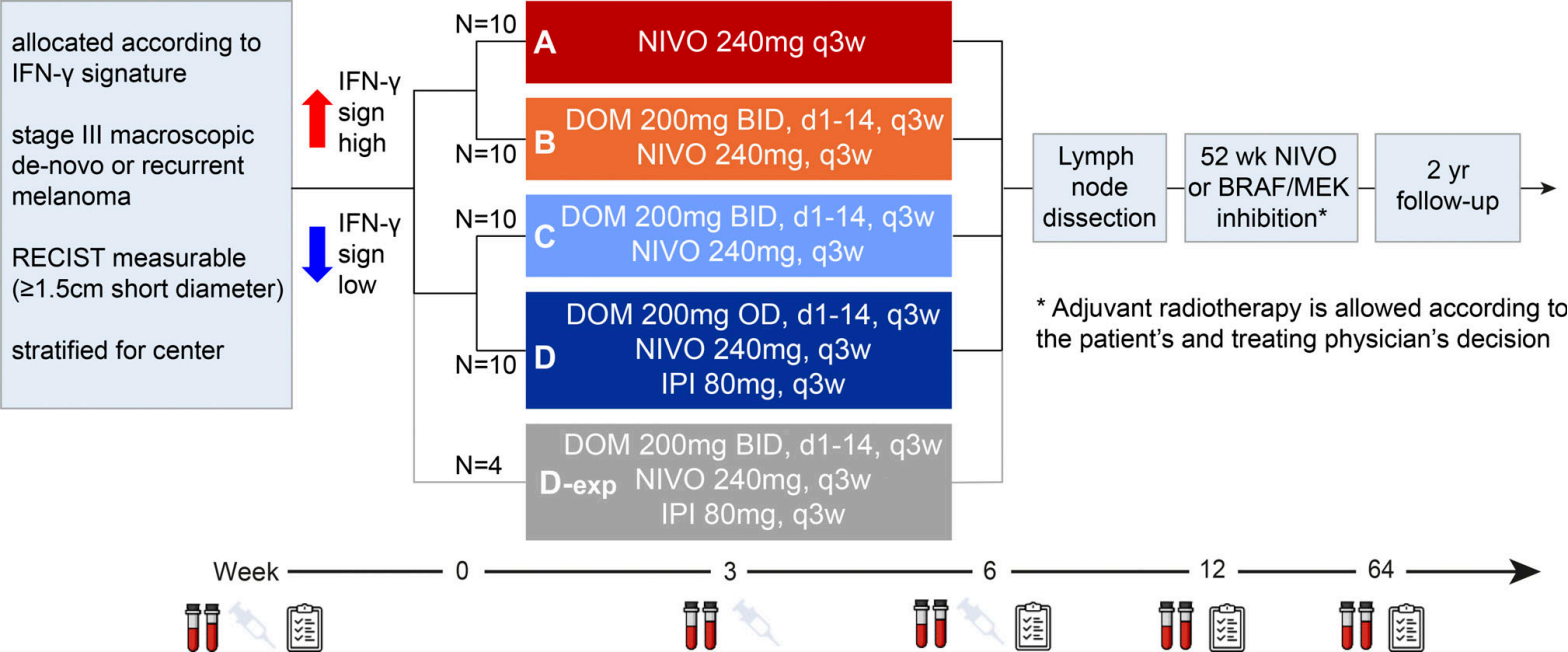
**DONIMI trial scheme.** Study design of the DONIMI trial. Asterisk indicates adjuvant radiotherapy according to patients and physicians’ decisions. DOM, domatinostat; IFN-γ sign, IFN-γ signature; IPI, ipilimumab; NIVO, nivolumab.

**Figure S2.**
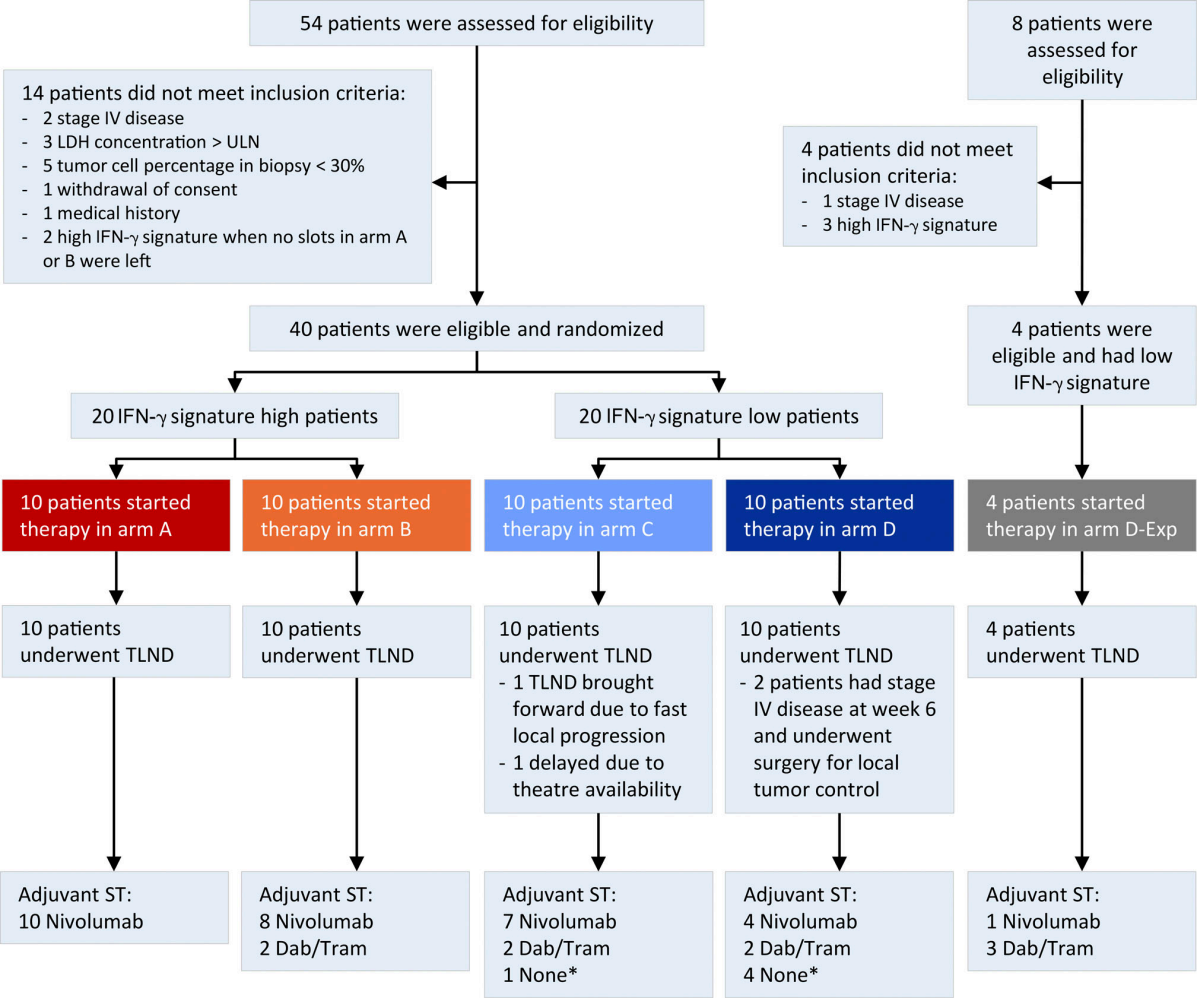
**Flow chart of the DONIMI trial.** Asterisk indicates that five patients did not receive adjuvant systemic therapy due to progression to stage IV disease (*n* = 3), toxicity during neoadjuvant treatment (*n* = 1), or patient’s choice (*n* = 1). Dab, dabrafenib; ST, systemic therapy; TLND, total lymph node dissection; Tram, trametinib; ULN, upper limit of normal.

Between January 2020 and April 2021, 20 patients with a high IFN-γ score in the tumor and 20 patients with a low IFN-γ score in the tumor were enrolled and randomized to arms A or B and C or D, respectively (Fig. 2 and Fig. S2). Clinical baseline characteristics were comparable between arm A versus B and C versus D, except for a higher percentage of BRAF^V600E/K^ mutated tumors in arm D (80%) compared to arm C (30%; Table 1). Baseline IFN-γ scores of patients comparing arm A versus B (P = 0.227) and C versus D (P = 0.697) were similar. At data cut-off (July 24, 2022), the median follow-up was 19.7 (IQR 19.2–22.4) mo from randomization with a minimum follow-up of 8.4 mo for patients who were alive.

**Table 1.** Baseline characteristics

Characteristic	A: IFN-γ high NIVO (*n* = 10)		B: IFN-γ high NIVO + DOM BID (*n* = 10)		C: IFN-γ low NIVO + DOM BID (*n* = 10)		D: IFN-γ low IPI + NIVO + DOM QD (*n* = 10)		D-exp: IFN-γ low IPI + NIVO + DOM BID (*n* = 4)	
Age, median (range)	56.5	(36–69)	60.5	(33–81)	61	(26–76)	62	(37–80)	58.5	(53–69)
Sex, male	5	(50%)	5	(50%)	8	(80%)	8	(80%)	2	(50%)
WHO performance status, 0	10	(100%)	10	(100%)	9	(90%)	8	(80%)	3	(75%)
**Primary tumor**										
Breslow thickness, median (range)	16	(0.1–9)	1.3	(0.5–4)	2.2	(0.8–15)	1.2	(0.6–2.1)	3.0	(0.7–3.0)
Ulceration	2	(20%)	3	(30%)	4	(40%)	0	(0%)	0	(0%)
Unknown primary	1	(10%)	1	(10%)	2	(20%)	4	(40%)	1	(25%)
**Location lymph node metastases**										
Neck	0	(0%)	0	(0%)	4	(40%)	1	(10%)	3	(75%)
Axilla	3	(30%)	6	(60%)	3	(30%)	5	(50%)	0	(0%)
Inguinal/iliac region	7	(70%)	4	(40%)	3	(30%)	4	(40%)	1	(25%)
Sum target lesions on CT, median (range)	19.5	(15–33)	28.5	(15–67)	17.5	(15–32)	29.5	(16–86)	21.5	(18–28)
LDH, <ULN	10	(100%)	10	(100%)	10	(100%)	10	(100%)	4	(100%)
BRAF^V600E/K^ positive	4	(40%)	6	(60%)	3	(30%)	8	(80%)	3	(75%)

Data are median (range) or *n* (%). DOM, domatinostat; IPI, ipilimumab; NIVO, nivolumab; ULN, upper limit of normal.

#### Feasibility and toxicity

All patients in arms A, B, and C received the two planned neoadjuvant cycles of nivolumab, while in arm D, 80% of patients received the two planned cycles of nivolumab and ipilimumab (one patient with grade 3 immunotherapy-related nephritis and one with grade 2 myalgia and arthralgia received only one cycle; Table 2). The planned domatinostat dosing was given in 60% of patients in arm B, 40% of patients in arm C, and 80% of patients in arm D. Three patients in arm B and four patients in arm C received only one cycle of domatinostat due to development of a domatinostat-related skin rash (at least grade 3 in six patients), and two patients in arm D with the above-described immunotherapy-related toxicities received only one cycle of domatinostat. Furthermore, one patient in arm B mistakenly took a lower domatinostat dose during the first cycle, and in two patients in arm C the domatinostat dose was reduced and interrupted due to grade 2 headaches. All patients underwent surgery within the preplanned time period (week 6 ± 1 wk) except for one patient in arm B for whom surgery was delayed due to theatre unavailability (Table 2).

**Table 2.** Treatment disposition

Treatment disposition	A: IFN-γ high NIVO (*N* = 10)		B: IFN-γ high NIVO + DOM BID (*N* = 10)		C: IFN-γ low NIVO + DOM BID (*N* = 10)		D: IFN-γ low IPI + NIVO + DOM QD (*N* = 10)		D-exp: IFN-γ low IPI + NIVO + DOM BID (*N* = 4)	
**Total cycles of NIVO**										
1	0	(0%)	0	(0%)	0	(0%)	2^A^	(20%)	0	(0%)
2	10	(100%)	10	(100%)	10	(100%)	8	(80%)	4	(100%)
**Total cycles of IPI**										
1	–	–	–	–	–	–	2^A^	(20%)	0	(0%)
2	–	–	–	–	–	–	8	(80%)	4	(100%)
ICI cycles delayed										
Due to trAE	0	(0%)	0	(0%)	2	(20%)	1	(10%)	2	(50%)
**Domatinostat administration**										
Completed w/o modifications	–	–	6	(60%)	4	(40%)	8	(80%)	0	(0%)
Interrupted	–	–	0	(0%)	2^D^	(20%)	0	(0%)	0	(0%)
Dose reduction	–	–	1^B^	(10%)	2^D^	(20%)	0	(0%)	0	(0%)
Discontinued prematurely	–	–	3 C	(30%)	4^C^	(40%)	2^A^	(20%)	4	(100%)
**Surgery**										
Performed	10	(100%)	10	(100%)	10	(100%)	10	(100%)	4	(100%)
Delayed	0	(0%)	1^E^	(10%)	0	(0%)	0	(0%)	0	(0%)
Brought forward	0	(0%)	0	(0%)	1^F^	(10%)	0	(0%)	0	(0%)
**Adjuvant therapy**										
Nivolumab	10	(100%)	8	(80%)	7	(70%)	4	(40%)	1	(25%)
Dabrafenib/trametinib	0	(0%)	2	(20%)	2	(20%)	2	(20%)	3	(75%)
Radiotherapy	1	(10%)	1	(10%)	4	(40%)	4	(40%)	2	(50%)

Data are *n* (%). (A) Only 1 cycle IPI + NIVO + DOM due to grade 3 ir-nephritis (*n* = 1) and grade 2 ir-myalgia/arthralgia (*n* = 1). (B) Patient mistakenly took DOM 200 mg QD during first cycle instead of BID. (C) Prematurely discontinuation due to grade 2–3 DOM-related rashes/hypersensitivity reaction. (D) DOM interrupted and dose reduced in two patients due to grade 2 headaches. (E) Surgery delayed due to theater availability. (F) Surgery was brought forward due to fast clinical progression. DOM, domatinostat; ICI, immune checkpoint inhibition; IPI, ipilimumab; NIVO, nivolumab.

**Figure 3.**
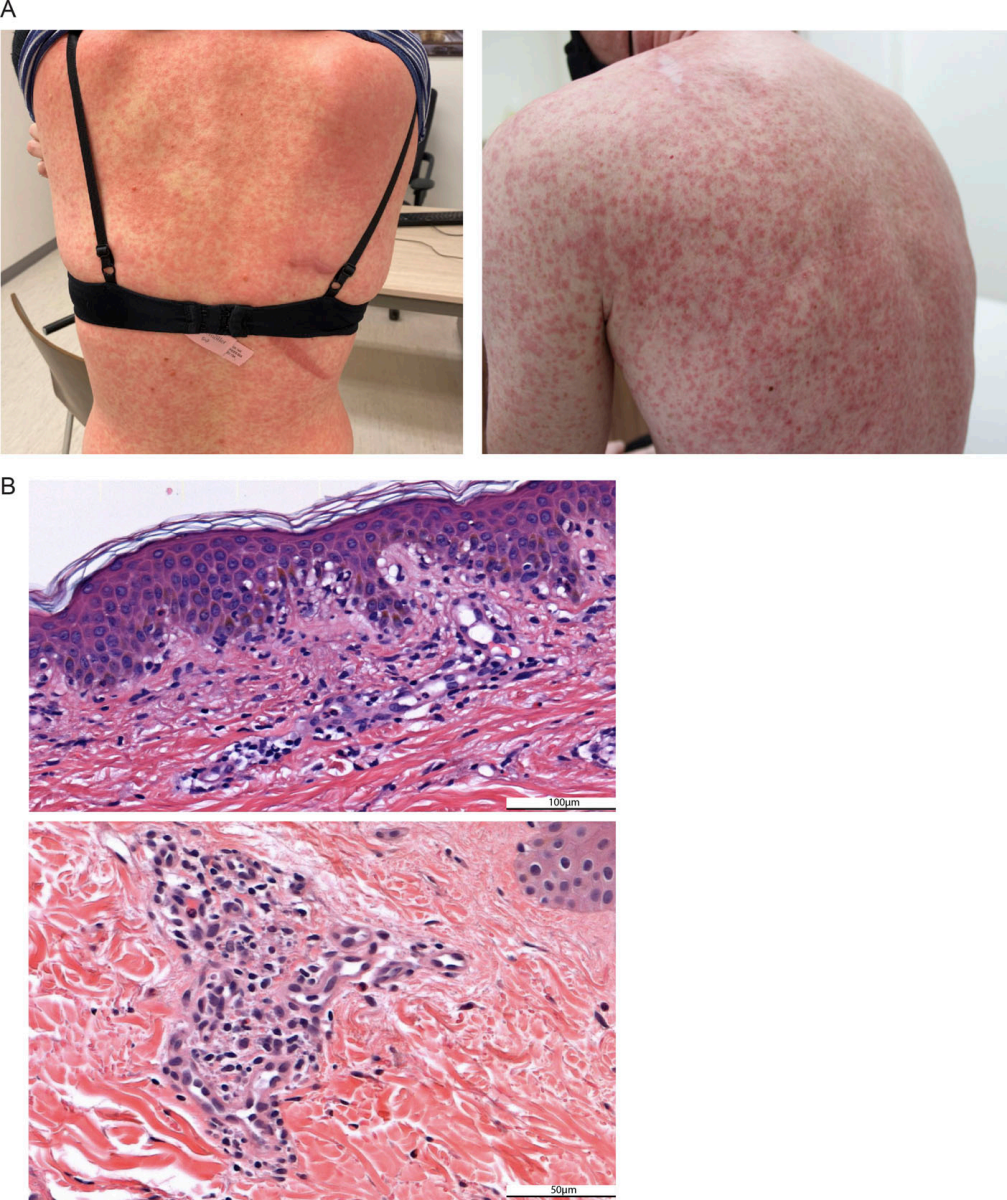
**Domatinostat-related skin toxicity. (A)** Photos of domatinostat-related skin rash showing a generalized and partly confluent maculopapular rash. **(B)** Representative H&E sections of a patients’ skin biopsy taken during the skin rash showing a vacuolar interface dermatitis with apoptotic keratinocytes, a mild superficial perivascular lymphohistiocytic infiltrate with eosinophils, and in some cases extravasation of erythrocytes (i.e., small vessel vasculitis component). Scale bar is 100 μm (top) or 50 μm (bottom).

During the first 12 wk after initiation of neoadjuvant therapy, grade 3 drug treatment–related adverse events (trAEs) were observed in 0% in arm A, 20% in arm B, 40% in arm C, and 20% in arm D (Table 3). No grade 4 trAEs or treatment-related deaths occurred. The most prevalent grade 3 trAEs in arms B and C were skin rashes. The observed skin generalized maculopapular rashes typically had an onset after 10–12 d of treatment, spread rapidly covering >30% of body surface area, and were regularly accompanied with other symptoms like fever, malaise, headache, and pruritus (Fig. 3 A). On H&E skin sections, a dermal hypersensitivity reaction with a vacuolar pattern was found (Fig. 3 B), which is in some cases associated with signs of small vessel vasculitis. Skin toxicity was manageable with topical or systemic corticosteroids and permanent cessation of the domatinostat tablets. Rapid rash recurrence was seen in two patients who were retreated with domatinostat, whereas the rash did not recur when nivolumab only was continued, resembling a rapid onset of a delayed hypersensitivity-like reaction triggered by domatinostat. During the adjuvant treatment period, one additional patient (in arm A) developed grade ≥3 trAEs (Table S1, A and B).

**Table 3.** Systemic trAEs during the first 12 wk

Adverse event	A: IFN-γ high NIVO (*n* = 10)				B: IFN-γ high NIVO + DOM BID (*n* = 10)				C: IFN-γ low NIVO + DOM BID (*n* = 10)				D: IFN-γ low IPI + NIVO + DOM QD (*n* = 10)				D-exp: IFN-γ low IPI + NIVO + DOM BID (*n* = 4)			
	Grade 1–2		Grade 3		Grade 1–2		Grade 3		Grade 1–2		Grade 3		Grade 1–2		Grade 3		Grade 1–2		Grade 3	
Any adverse event	10	100%	0	0%	8	80%	2	20%	6	60%	4	40%	8	80%	2	20%	0	0%	4	100%
Skin rash	3	30%	0	–	3	30%	2	20%	3	30%	4	40%	2	20%	0	–	1	25%	3	75%
Fatigue	6	60%	0	–	2	20%	0	–	5	50%	0	–	6	60%	0	–	1	25%	0	–
Pruritus	3	30%	0	–	1	10%	0	–	3	30%	0	–	3	30%	0	–	3	75%	0	–
ALT increased	1	10%	0	–	2	20%	0	–	4	40%	1	10%	2	20%	0	–	2	50%	0	–
AST increased	1	10%	0	–	2	20%	0	–	4	40%	0	–	2	20%	0	–	2	50%	0	–
Headache	0	–	0	–	3	30%	0	–	4	40%	0	–	2	20%	0	–	2	50%	0	–
Dry mouth	1	10%	0	–	4	40%	0	–	1	10%	0	–	1	10%	0	–	1	25%	0	–
Fever	0	–	0	–	1	10%	0	–	3	30%	1	10%	1	10%	0	–	2	50%	0	–
Lipase increased	1	10%	0	–	3	30%	0	–	0	–	0	–	2	20%	0	–	1	25%	0	–
Myalgia	2	20%	0	–	0	–	0	–	1	10%	0	–	3	30%	0	–	1	25%	0	–
Nausea	0	–	0	–	2	20%	0	–	2	20%	0	–	3	30%	0	–	0	–	0	–
Arthralgia	2	20%	0	–	1	10%	0	–	0	–	0	–	2	20%	0	–	0	–	0	–
Infusion-related reaction	1	10%	0	–	1	10%	0	–	1	10%	0	–	2	20%	0	–	0	–	0	–
Amylase increased	0	–	0	–	2	20%	0	–	1	10%	0	–	2	20%	0	–	0	–	0	–
Diarrhea	0	–	0	–	0	–	0	–	0	–	0	–	3	30%	1	10%	0	–	0	–
Gastrointestinal pain	1	10%	0	–	1	10%	0	–	1	10%	0	–	1	10%	0	–	0	–	0	–
Platelet count decreased	0	–	0	–	0	–	0	–	1	10%	0	–	1	10%	0	–	2	50%	0	–
Hyperthyroidism	0	–	0	–	0	–	0	–	0	–	0	–	3	30%	0	–	0	–	0	–
Dysgeusia	0	–	0	–	0	–	0	–	2	20%	0	–	1	10%	0	–	0	–	0	–
Stomatitis	0	–	0	–	0	–	0	–	1	10%	0	–	0	–	0	–	0	–	1	25%
Acute kidney injury	0	–	0	–	0	–	0	–	0	–	0	–	0	–	1	10%	0	–	0	–

Data are *n* (%). trAEsthat occurred in ≥5% of patients, and all grade 3–4 are displayed in the table. Within the first 12 wk, no grade 4 or 5 adverse events were observed. ALT, alanine aminotransferase; AST, aspartate aminotransferase; DOM, domatinostat; IPI, ipilimumab; NIVO, nivolumab.

Surgical procedures performed after neoadjuvant therapy were axillary lymph node dissection, inguinal ± iliacal lymph node dissection or a modified radical neck dissection in 43, 45, and 13% of patients, respectively. Surgery-related grade 3 adverse events occurred in three patients (30%) in arm A, and in one (10%), one (10%), and two (20%) patients in arms B, C, and D, respectively (Table S1 C). The most prevalent surgery-related adverse events were seroma (70%), wound infection (43%), and wound dehiscence (23%).

#### Efficacy and survival

Radiologic objective responses at pre-surgical evaluation were observed in five (50%) patients in arm A, seven (70%) in arm B, no (0%) patient in arm C, and four (40%) patients in arm D (Table S2). Progressive disease was observed in one patient in arm C (local progression observed on MRI neck, the week 6 CT scan was not evaluable for response because surgery was brought forward due to impending irresectability) and five patients in arm D (three with local resectable progression and two with distant metastases). The two patients with distant metastases underwent surgery for local tumor control (*n* = 1) or because the metastasis was thought to be a benign lesion presurgically (*n* = 1). Histopathologic assessment of the week 6 resected surgical specimens revealed a pathologic response rate (≤50% residual viable tumor) of 90% in arm A, 80% in arm B (both arms included IFN-γ high patients), 30% in arm C, and 40% in arm D (both arms included IFN-γ low patients; Table 4). Pathologic response was not associated with domatinostat-related skin toxicity; the pathologic response rate was 54% (7/13 patients) for patients with skin toxicity and 59% (26/44) for the total cohort.

**Table 4.** Pathological response according to the INMC criteria

Pathological response	A: IFN-γ high NIVO (*n* = 10)		B: IFN-γ high NIVO + DOM BID (*n* = 10)		C: IFN-γ low NIVO + DOM BID (*n* = 10)		D: IFN-γ low IPI + NIVO + DOM QD (*n* = 10)		D-exp: IFN-γ low IPI + NIVO + DOM BID (*n* = 4)	
pRR 95% confidence interval (0–50% viable tumor)	9	(90%) (55–99.7%)	8	(80%) (44–97%)	3	(30%) (7–65%)	4	(40%) (12–74%)	2	(50%) (7–93%)
pCR (0% viable tumor)	8	(80%)	4	(40%)	1	(10%)	3	(30%)	–	–
near-pCR (>0–10% viable tumor)	–	–	2	(20%)	–	–	1	(10%)	1	(25%)
pPR (>10–50% viable tumor)	1	(10%)	2	(20%)	2	(20%)	–	–	1	(25%)
pNR (>50% viable tumor)	1	(10%)	2	(20%)	7	(70%)	6	(60%)	2	(50%)

DOM, domatinostat; IPI, ipilimumab; NIVO, nivolumab; pCR, pathological complete response; pNR, pathological non-response; pPR, pathological partial response; pRR, pathological response rate.

**Figure 4.**
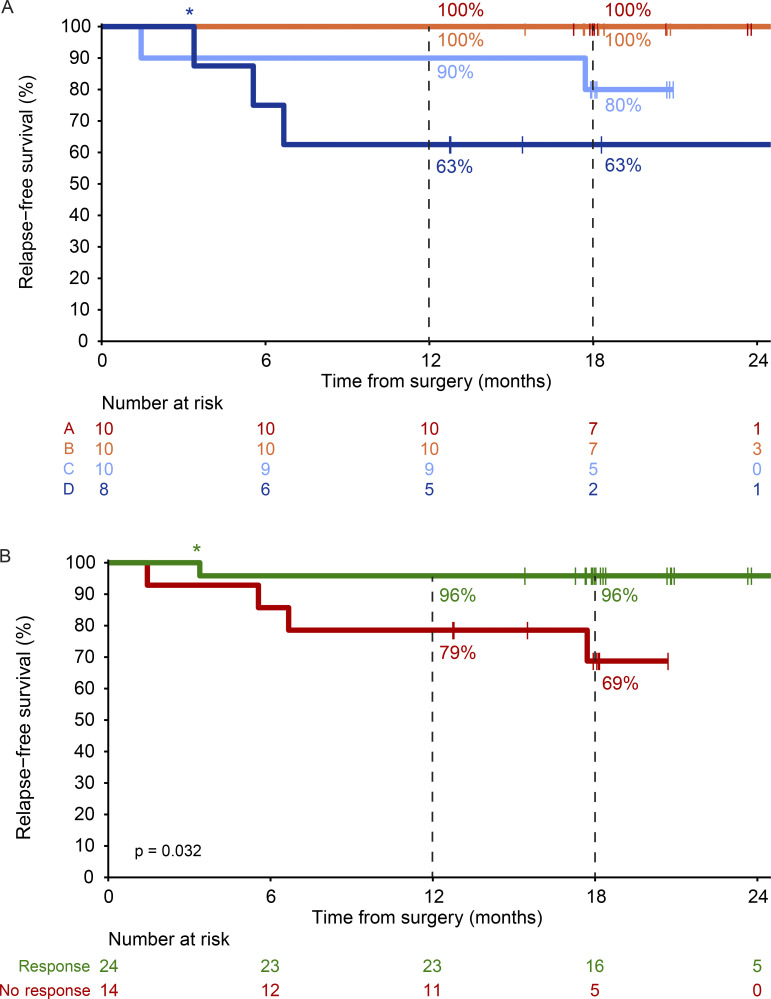
**RFS. (A)** RFS by treatment arm of patients of arm A–D (*n* = 38). Two patients from arm D who had progression prior to surgery were excluded. **(B)** RFS of patients from A to D by pathologic response (*n* = 38). Asterisk indicates that the death of this patient was not melanoma- or treatment-related.

**Figure S3.**
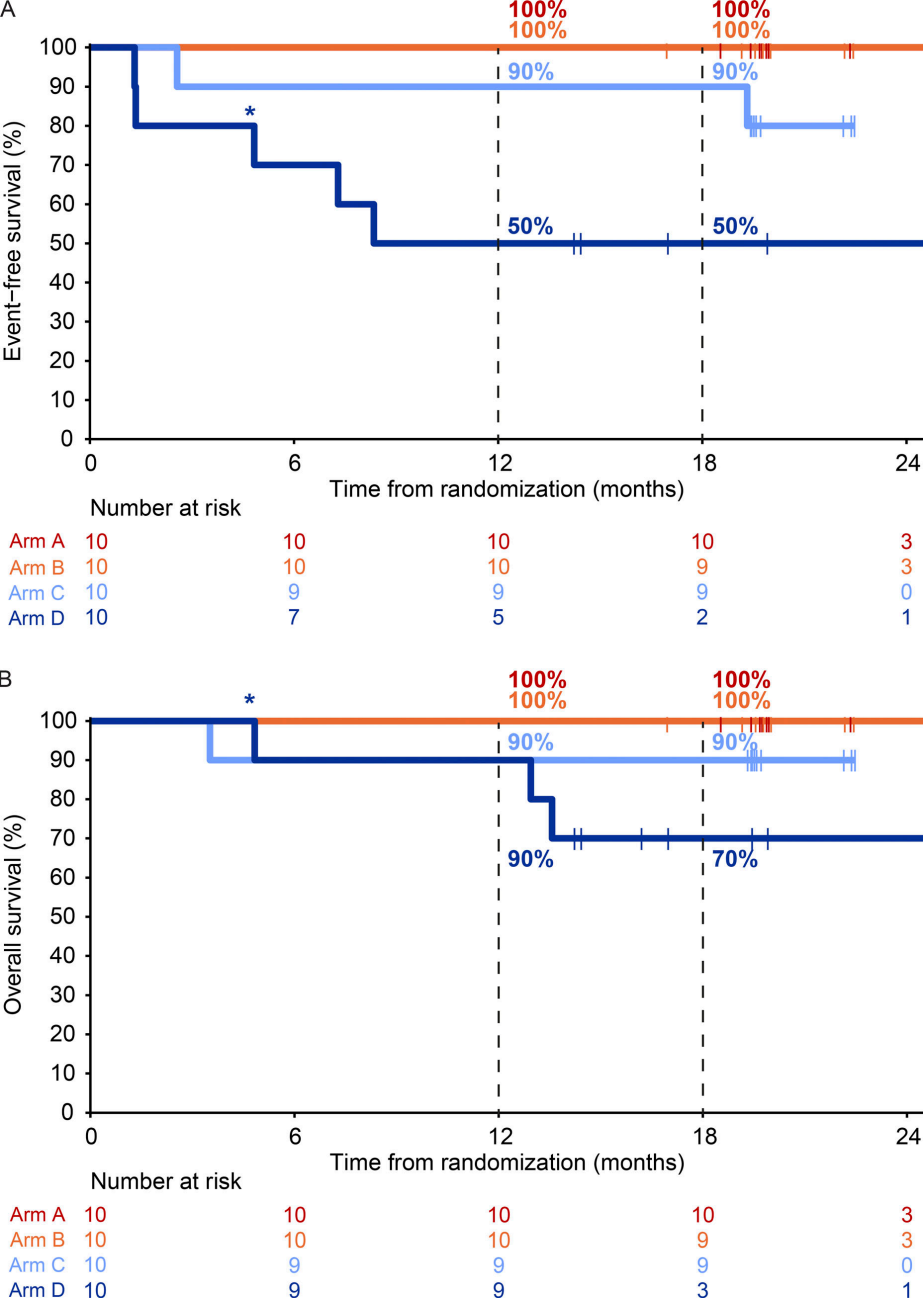
**Event-free and overall survival according to treatment arm (A–D). (A)** EFS per treatment arm of the DONIMI trial (arm A–D, each *n* = 10). **(B)** OS per treatment arm of the DONIMI trial (arm A–D, each *n* = 10). Asterisk indicates that the death of this patient was not melanoma- or treatment-related.

Adjuvant systemic therapy with either nivolumab or dabrafenib + trametinib (only for patients with a pathologic non-response and a BRAFV600E/K mutated melanoma) was commenced from week 12 for a period of 52 wk. Adjuvant radiotherapy was also allowed in patients without pathologic response (Table 2). In total, 5/40 (13%) patients did not receive adjuvant systemic therapy; three due to progression to stage IV disease, one due to toxicity during neoadjuvant treatment, and one patient choice. At data cutoff, the median RFS was not reached for any of the arms (Fig. 4 A). The estimated 18-mo RFS rate was 100% in arm A and B, 80% (95% CI 59–100%) in arm C, and 63% (95% CI 37–100%) in arm D (Fig. 4 A). Improved 18-mo RFS was observed in patients with pathologic response (96%, 95% CI 88–100%) versus those without response (69%, 95% CI 47–100%, P = 0.032; Fig. 4 B). Median event-free survival (EFS) was 8.3 mo for arm D, and not reached for arms A, B, and C (Fig. S3 A). At data cutoff, one patient in arm C and three patients in arm D had died, including one patient in arm D who died without evidence of melanoma recurrence (Fig. S3 B).

#### Arm D expansion

Between July 2021 and November 2021, four patients were enrolled in an expansion cohort of arm D (D-exp), testing domatinostat at an escalated dose of 200 mg twice daily (BID) in combination with ipilimumab + nivolumab. Baseline characteristics are shown in Table 1. All four patients developed the domatinostat-related skin toxicity (75% grade 3), requiring premature discontinuation of domatinostat, administration of oral prednisone, and delay of the second ipilimumab + nivolumab cycles in two patients (Tables 2 and 3). One patient simultaneously developed grade 3 stomatitis. All patients underwent surgery on time, and two patients achieved a pathologic response (pRR 50%, 95% CI 7–93%; Table 4). Although arm D-exp met the feasibility criterion of no delays of surgery, the data safety monitoring board (DSMB) recommended cessation of further recruitment given no patients were able to complete the neoadjuvant domatinostat treatment course.

#### Biomarker analyses for response

**Figure 5.**
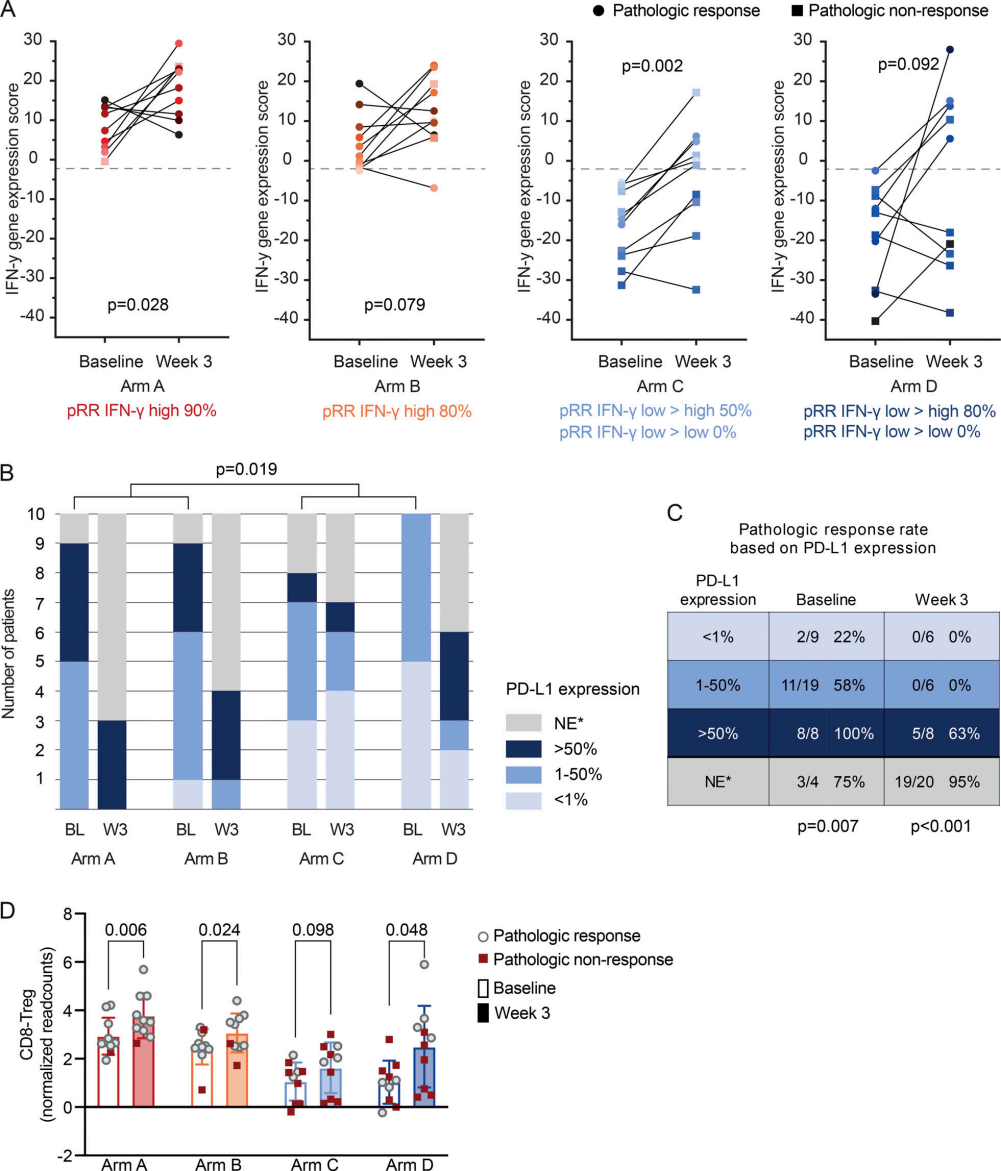
**Biomarker analyses for response. (A)** The IFN-γ gene expression score at baseline and week 3 across treatment arms, calculated with normalized NanoString readcounts. Baseline versus on-treatment IFN-γ scores were compared using two-tailed paired *t* tests. Pathologic responders are indicated with circles, and pathologic non-responders with squares. **(B and C)** Frequency of the PD-L1 tumor proportion score categories at baseline and week 3 (B) and pathologic response rates per category (C). Baseline PD-L1 scores of arm A + B versus C + D were compared using χ-square test, and association between PD-L1 score and response was calculated with two-tailed Fisher’s exact test. *, NE = not evaluable, mainly due to the absence of tumor or high pigmentation. **(D)** Normalized NanoString readcounts indicating ratio of CD8^+^ T cells and FOXP3^+^ Tregs in patient tumors at baseline and week 3 across treatment arms. Statistical significance across baseline and week 3 within each arm was evaluated using two-tailed paired Student’s *t* test. pRR, pathologic response rate.

We examined several biomarkers to evaluate the potential immune-modulating effect of domatinostat and associations with pathologic response. Paired baseline and on-treatment biopsies of patients from arm A to D were analyzed for the presence and changes of the IFN-γ score using NanoString panCancer Immunology Panel analysis (Fig. 5 A). All arms showed (a trend towards) an increased mean IFN-γ score on-treatment. However, the magnitude of increase was not higher in arm B (nivolumab + domatinostat, mean IFN-γ score increase 7.51) compared to arm A (nivolumab monotherapy, mean IFN-γ score increase 9.87). Notably, in a subset of patients with a low baseline intratumoral IFN-γ score (arms C and D) the signature gene expression was converted to a high IFN-γ score after 3 wk of treatment (“IFN-γ low→high scores”), which resulted in a pathologic response in 3/6 (50%) patients in arm C and 4/5 (80%) patients in arm D. All patients in arm C and D in which the on-treatment IFN-γ score remained low (“IFN-γ low→low scores”) were pathologic non-responders at week 6 (Fig. 5 A). The baseline and on-treatment IFN-γ scores were not associated with domatinostat-related skin toxicity (data not shown).

Exposure of a tumor cell to IFN has been previously shown to induce PD-L1 expression on tumor cells (Latchman et al., 2001; Blank et al., 2006). In line with this, the baseline PD-L1 expression on tumor cells was higher in patients in arms A and B versus patients in arms C and D (P = 0.019), and was associated with the baseline IFN-γ score (P < 0.001; Fig. 5 B). On treatment, the PD-L1 expression increased for most patients or was not evaluable due to the absence of tumor and/or pigmentation (Fig. 5 B). The PD-L1 scores at baseline and week 3 were associated with pathologic response (P = 0.007 and P < 0.001, respectively; Fig. 5 C). In line with the IFN-γ low→low expression scores, all patients who had a PD-L1 score of ≤50% at week 3 had a pathologic non-response after 6 wk (Fig. 5 C).

There was an increased CD8/Treg ratio upon domatinostat treatment in our preclinical murine melanoma model (Fig. 1 D). Hence, to evaluate if this occurred also in the patients treated with domatinostat, the ratio of CD8^+^ T cells to FoxP3^+^ Tregs in the tumor was examined using NanoString. The baseline results indicated a clear distinction between the CD8/Treg ratios in patients across treatment arms, with baseline CD8/Treg ratio in most patients in arms C and D being lower than the mean CD8/Treg ratio of patients in arms A and B, suggesting that patients with low IFN-γ score also had lower CD8/Treg ratio (Fig. 5 D). We observed a significant increase in the CD8/Treg ratio upon treatment in arms A, B, and D, and a trend for increased ratio in arm C (Fig. 5 D). However, and similar to the IFN-γ scores, domatinostat did not cause a greater increase in the CD8/Treg ratio in arm B (mean CD8/Treg ratio increase 0.574) compared to arm A (mean CD8/Treg ratio increase 0.842). The baseline and on-treatment CD8/Treg ratios were also comparable between patients with and without domatinostat-related skin toxicity (data not shown).

In summary, these data indicate that, in contrast to our preclinical data, the addition of domatinostat (at the given doses) to neoadjuvant ipilimumab ± nivolumab did not result in improved immunomodulation.

## Discussion

To our knowledge, DONIMI is the first neoadjuvant immunotherapy trial that used a biomarker to allocate therapy in melanoma by prospectively testing an IFN-γ signature gene expression score to allocate patients with stage III melanoma to different combinations of neoadjuvant ICB with domatinostat. The trial design was informed by results from our preclinical melanoma tumor model that showed enhanced tumor growth control, prolonged survival of mice and an increased IFN-γ response upon the combination of anti-PD-1 + anti-CTLA-4 with domatinostat. The DONIMI trial showed that the combination of neoadjuvant ICB with domatinostat was feasible and that the IFN-γ signature score in a baseline tumor biopsy was associated with pathologic response to neoadjuvant ICB. Patients with a high IFN-γ score had higher pathologic response rates (despite treatment de-escalation) compared to patients with a low score.

This IFN-γ signature–driven approach was based on results from our previous neoadjuvant trials that showed that the baseline IFN-γ score was a predictive biomarker for pathologic response (Rozeman et al., 2021). RNA expression analysis using NanoString nCounter technologies of patient cohorts from our OpACIN-neo and PRADO studies showed a pathologic response rate of 89% for patients with a high IFN-γ score in their baseline tumor biopsy versus 49% for patients with a low IFN-γ score (Reijers et al., 2022a). These data, together with the strong association between pathologic response and long-term RFS (Menzies et al., 2021), highlighted the possibility of de-escalating neoadjuvant therapy in patients with a high IFN-γ score, and emphasized the need for more effective neoadjuvant treatment combinations for patients with low IFN-γ scores in their tumor.

While we have not fully excluded the possibility of mild subclinical dermatitis that might have been concealed by the fur, we did not observe any visible skin toxicities (such as signs of alopecia, loss of whiskers/irritated skin on face and limbs, excessive grooming, or bad skin condition) in our murine model upon treatment with domatinostat. In contrast to the results of our preclinical melanoma model and the SENSITIZE trial testing domatinostat + pembrolizumab in stage IV melanoma (Hassel et al., 2021), the combination of domatinostat with neoadjuvant nivolumab ± ipilimumab led to unexpected frequent and intense grade 3 skin toxicities in our patients with stage III melanoma, especially with the 200 mg BID dosing scheme. Although the exact mechanism is unknown, the skin toxicity is thought to be a hypersensitivity reaction triggered by domatinostat, as re-challenge with only ICB did not result in reoccurrence, but re-challenge with domatinostat caused a rapid reoccurrence of the rash within hours. This toxicity mandated early cessation of domatinostat in a substantial proportion of patients and prevented further investigation of the higher domatinostat dose combined with nivolumab + ipilimumab in patients with a low baseline IFN-γ score (arm D-exp).

Furthermore, the addition of domatinostat did not seem to increase the pathologic response rate of the neoadjuvant therapy for patients with a high IFN-γ score (80 versus 90% for nivolumab monotherapy) or with a low IFN-γ score (40–50 versus 49% seen in the historical data for ipilimumab + nivolumab; Reijers et al., 2022a). Both the studies in preclinical tumor models (including our MeVa2.1.dOVA melanoma model) and in patients with advanced melanoma showed an increase in the expression of the IFN-γ signature genes and the CD8/Treg ratio with domatinostat treatment (Fig. 1 D; Bretz et al., 2019; Hassel et al., 2021). These immunomodulating effects of domatinostat were not corroborated by the patients in the DONIMI trial. Of note, although our murine flow cytometry analysis indicated a shift toward M2-like macrophages in the myeloid compartment, concurrent murine RNA sequencing data showed a clear increase in pro-inflammatory gene expression pathways. Therefore, we did not follow-up on this in patients. Whether the inability to administer the two complete cycles of domatinostat in the recommended phase 2 dose (especially for patients treated with the triple combination) also contributed to the lack of effect remains elusive.

A major finding of DONIMI is that patients with a high IFN-γ score can achieve high pathologic response rates with nivolumab monotherapy, thus might not need treatment with ipilimumab + nivolumab or other combinations, e.g., nivolumab + relatimab (anti-LAG-3 antibody; Amaria et al., 2022). The pathologic response rate in patients with a high IFN-γ score treated with nivolumab (90%) was comparable to response rates observed in patients with a high IFN-γ score treated with combined ipilimumab and nivolumab (89%; Reijers et al., 2022a). Previous trials testing neoadjuvant anti-PD-1 monotherapy in an unselected population showed pathologic response rates of only 30–55% (Huang et al., 2019; Amaria et al., 2018; Long et al., 2022), suggesting that the IFN-γ signature may be an effective biomarker for patient selection.

Our data indicate that the concept of tumor immune inflammation by division into immune infiltrated, immune excluded, and immune desert tumors (Chen and Mellman, 2013) could also be interpreted as a dynamic rather than a static situation. Hence, additional benefit might arise from on-treatment evaluation of the IFN-γ score, as patients with a low baseline IFN-γ score that became high early on-treatment (IFN-γ low→high scores) achieved pathologic responses in 50% in arm C (nivolumab + domatinostat) and 80% in arm D (ipilimumab + nivolumab + domatinostat), whereas patients with IFN-γ low→low scores had a 0% pathologic response rate. Therefore, we hypothesized that patients with a high baseline IFN-γ score might obtain long-term benefit from anti-PD-1 monotherapy, patients with IFN-γ low→high score can continue with a second dose ipilimumab + nivolumab, whereas patients with an IFN-γ low→low score are in need of alternative drugs prior to surgery.

Biomarker analyses from larger trials testing neoadjuvant anti-PD-1 monotherapy are needed to confirm the high pathologic response rates of the DONIMI patients with a high IFN-γ score. This could be assessed in the phase II SWOG S1801 trial (NCT03698019) that tested neoadjuvant plus adjuvant pembrolizumab compared to adjuvant pembrolizumab in resectable stage III–IV melanoma and recently demonstrated an EFS benefit of 23% at 2 yr for the neoadjuvant arm (Patel et al., 2023).

One of the limitations of our murine study was that this was not conducted in a neoadjuvant setting, making direct comparisons of pathological responses between the murine model and the clinical trial challenging.

The DONIMI trial was limited by its small sample sizes. Therefore, our trial can only be seen as hypothesis-generating. Moreover, DONIMI did not randomize patients to the combination of ipilimumab + nivolumab, which is currently the most widely investigated neoadjuvant treatment regimen. Thereby, it allows for only indirect comparisons to historical cohorts with regard to efficacy and the additive effect of domatinostat for patients with a low baseline IFN-γ score treated with combination ICB.

Future clinical trials in stage III melanoma should focus on alternative (triple) treatment combinations for patients with an IFN-γ low→low score who are less likely to respond to ipilimumab + nivolumab. Combined nivolumab + relatlimab has shown promising efficacy with tolerable toxicity in advanced melanoma and as neoadjuvant therapy in stage III melanoma (Tawbi et al., 2022; Amaria et al., 2021). Hence, ipilimumab, nivolumab, and relatlimab could be a reasonable combination for these patients.

## Materials and methods

### MeVa2.1.dOVA melanoma tumor model

#### Mice strains

Immune-competent C57BL/6JRj mice were purchased from Janvier, and immune-compromised NOD-scid IL2rγ^null^ (NSG) mice were bred and maintained at the animal facility of the Netherlands Cancer Institute (NKI, Amsterdam, Netherlands) under standard housing conditions. All female mice were used at the age of 8–9 wk. The animal experiments were performed in accordance with institutional and national guidelines and were approved by the Experimental Animal Committee of the NKI.

#### In vivo tumor growth

The MeVa2.1.dOVA cell line, established in our group (Rao et al., 2023), was cultured in advanced DMEM/F-12 (cat: 12634028; Thermo Fisher Scientific) supplemented with heat-inactivated fetal bovine serum (cat: 3101517; Capricorn Scientific) and L-glutamine (cat: 25030081; Thermo Fisher Scientific). Routine testing of the cell line was performed to ensure cells remained negative for mycoplasma. 0.3 million cells in 50 μl were mixed with equal volume of Matrigel (cat: 354234; Corning) and injected subcutaneously into the shaven right flank of mice under anesthesia (isoflurane + oxygen). Two-dimensional calliper measurements of the greatest longitudinal and transverse diameters were used to obtain tumor volume as per modified ellipsoidal formula: tumor volume = length × width × (width/2). Mice were randomized into treatment arms based on tumor volume when the average tumor size of the group was between 100 and 150 mm^3^. Domatinostat (formulation: DMSO in cremaphor + water, 4SC) was given BID via oral gavage (20 mg/kg, at least 8 h gap between two doses, for a total of 28 cycles). Anti-PD-1 (100 μg/dose; clone RMP1-14, cat: BE0146; BioXCell) ± anti-CTLA-4 (50 μg/dose; clone: 9D9, cat: BE0164; BioXCell) were injected twice weekly intraperitoneally. Treatment lasted for 14 d. For experiments to monitor the effect on tumor outgrowth, mice were sacrificed at tumor volume ≥1,500 mm^3^. However, if tumors that were smaller than 500 mm^3^ developed ulceration that did not heal within 3 d or if the mice had poor health state, unrelated to the treatments given, such mice were sacrificed before the experimental endpoint and not included for analysis. For flow cytometry analysis, mice were sacrificed after 13 d of treatment. Mice were euthanized at the endpoint using excess CO_2_.

#### Flow cytometry analysis

Single-cell suspensions of tumors were obtained by enzymatic digestion for 1 h in media containing collagenase A (cat: 11088793001, Roche) and DNAse (cat: 4716728001; Sigma-Aldrich). Fc blocking was performed using CD16/CD32 monoclonal antibody (clone: 93, cat: 14-0161-85; eBioscience) followed by cell surface staining with fluorochrome conjugated antibodies. Dead cells were excluded by staining with LIVE/DEAD Fixable Yellow Dead Cell Stain Kit (cat: L34968; Thermo Fisher Scientific). Intracellular staining was performed after permeabilization using Intracellular Fixation & Permeabilization Buffer Set (cat: 00552100; eBioscience) as per the manufacturer’s instructions.

The following antibodies were used: anti-mouse CD45.2 (clone: 104, cat: 560696) and anti-mouse CD11b (clone: M1/70; cat: 557396) were from BD Biosciences, anti-mouse CD3 (clone: 145-2C11; cat: 11-0031-85), anti-mouse CD8a (clone: 53-6.7; cat: 46-0081-82), anti-mouse FoxP3 (clone: FJK-16 s, cat: 17-5773-82), anti-mouse MHC II (clone: M5/114.15.2, cat: 46-5321-82), and anti-mouse CD206 (clone: MR6F3, cat: 12-2061-82) were from eBioscience, and anti-mouse CD4 (clone: GK1.5, cat: 100453) and anti-mouse F4/80 (clone: BM8, cat: 123118) were from BioLegend, respectively. H2Kb SIINFEKL tetramer was a kind gift from Ton N. Schumacher (NKI, Amsterdam, Netherlands) and described previously (Toebes et al., 2009). Samples were acquired in LSR Fortessa (BD Biosciences) and analyzed using FlowJoTM (v10.7.1, BD Life Sciences).

#### RNA sequencing and analysis

MeVa2.1.dOVA tumor–bearing mice were sacrificed after 13 d of treatment and tumors harvested and snap-frozen for RNA sequencing. RNA extraction and sequencing was performed by CeGat. RNA was isolated from samples using RNeasy Mini kit (Qiagen) as per the manufacturer’s instructions. Quality and quantity of the total RNA was assessed by Qubit (Thermo Fisher Scientific) and Bioanalyzer (Agilent). Total RNA samples having RIN >8 were subjected to library generation. Strand-specific libraries were generated using the TruSeq Stranded mRNA sample preparation kit (Illumina Inc.) according to the manufacturer's instructions. The libraries were sequenced with NovaSeq 6000. Demultiplexing of the sequencing reads was performed with Illumina bcl2fastq (2.20). Adapters were trimmed with Skewer (v 0.2.2). The quality of the FASTQ files was analyzed with FastQC (v 0.11.5-cegat). The samples were mapped with STAR (v 2.7.3a) to mouse reference genome Mus_musulus.GRCm39.105 using default settings. The read counts were computed with HTseq-count (v 0.12.4) and were analyzed with DESeq2 (v 1.30.1). Centering of the normalized gene expression read counts was performed by subtracting the row means and scaling by dividing the columns by the SD to generate a z-score. Gene set enrichment analysis was performed using GSEA (v 4.2.3, Broad Institute).

#### Statistical analysis

Tumor sizes across groups were compared at the last time point at which mice across all groups were alive. This was on day 20 and day 32 after tumor injection for C57BL/6 and NSG mice, respectively. Groups were compared using one-way ANOVA, followed by correction for multiple comparisons using Sidak’s test. For comparing two groups only, an unpaired Student’s *t* test was used. Kaplan-Meier plots were used to indicate survival and groups were compared using a log-rank test. P value <0.05 was considered statistically significant. All tests were performed using GraphPad Prism (v 9.3.1).

### The DONIMI trial

#### Study population

Patients were included in the NKI and Melanoma Institute Australia (MIA, Sydney, Australia). Eligible patients were ≥18 yr and had resectable stage III melanoma of cutaneous or unknown origin with at least one lymph node metastasis measurable according to RECIST v 1.1, a World Health Organization performance status of 0 or 1, and normal lactate dehydrogenase (LDH) levels. Key exclusion criteria included a history of in-transit metastases within 6 mo prior to inclusion, autoimmune diseases, and prior treatment with checkpoint inhibitors targeting CTLA-4, PD-1 or PD-L1, BRAF ± MEK inhibition, or radiotherapy.

#### Study design

The DONIMI trial is an investigator-initiated phase 1b trial that tested the feasibility and safety of neoadjuvant combinations of nivolumab ± ipilimumab with domatinostat according to the IFN-γ score in the tumor. Patients with a high IFN-γ score in their baseline tumor biopsy (*n* = 20) were randomized to arm A (two cycles nivolumab 240 mg every 3 wk) or arm B (two cycles nivolumab 240 mg + domatinostat 200 mg BID, day 1–14, every 3 wk). Patients with a low IFN-γ score (*n* = 20) were randomized to arm C (two cycles nivolumab 240 mg + domatinostat 200 mg BID, day 1–14, every 3 wk) or arm D (two cycles nivolumab 240 mg + ipilimumab 80 mg + domatinostat 200 mg once daily [QD], day 1–14, every 3 wk). After inclusion of the first 40 patients, 4 extra patients with a low IFN-γ score were included in an upfront planned expansion cohort of arm D (“arm D-exp”) and treated with the recommended phase 2 dose of domatinostat (two cycles nivolumab 240 mg + ipilimumab 80 mg + domatinostat 200 mg BID, day 1–14, every 3 wk). Lymph node dissection was planned 6 wk after the start of neoadjuvant treatment. At week 12, patients started with standard adjuvant nivolumab 480 mg every 4 wk or dabrafenib + trametinib (for patients with a pathologic non-response and BRAFV600E/K-mutated melanoma only) for 1 yr.

#### Randomization and blinding

Patients were stratified according to center and randomly assigned in a 1:1 ratio to arm A or B in case of a high IFN-γ score, and to arm C or D in case of a low IFN-γ score, using a block randomization scheme with ALEA software. The four patients in arm D-exp were not randomized. There was no blinding.

#### Endpoints and statistics

The primary endpoint of this trial was safety and feasibility of different combinations of neoadjuvant nivolumab ± ipilimumab with domatinostat, as measured by surgery timelines. Surgery was not on-time if it was not performed at week 6 ± 1 wk due to trAEs. Feasibility was monitored with stopping boundaries using the Bayesian method. For this, the maximum allowed probability of delayed surgery (p0) was set to 20% and the prior distribution of that probability to the non-informative β (0.5, 0.5). A treatment arm was deemed unfeasible if there was at least 70% probability that p0 >20%. The data were evaluated with the DSMB after inclusion of 5 and 10 patients in each arm. In case of two delays out of five patients or three delays out of 10 patients, the treatment arm would stop for safety. These stopping boundaries were calculated using the software for Bayesian toxicity monitoring at https://www.trialdesign.org from MD Anderson Cancer Center.

Secondary endpoints were pathologic response rate, radiologic response rate, all grade and grade 3–4 trAEs, RFS, and quality of life. Translational endpoints included identification and comparison of RNA signatures associated with pathologic response, changes in immune infiltrate, expansion of tumor-resident T cell clones, and feces microbiome analyses. The pathologic and radiologic responses as well as adverse events were summarized as proportions among all patients in treatment arm, and their corresponding two-sided 95% confidence intervals were calculated using the Clopper-Pearson method. Survival curves for every treatment arm were generated using the Kaplan-Meier method, and overall comparisons across arms were done with the log-rank test. For confidence intervals of survival rates, the standard error was computed using Greenwood’s formula. No adjustments for multiple comparisons were performed. Median follow-up was computed using the reverse Kaplan-Meier method. Analyses were performed using R (v 3.5.1).

#### Study assessments

All patients underwent baseline tumor staging using a CT scan of neck/thorax/abdomen/pelvis, a PET scan, and a cerebral MRI scan. Additionally, CT scans were conducted presurgically (week 5–6), before initiation of adjuvant treatment (week 12), every 12 wk thereafter until 3 yr from randomization, and in year 4 and 5 according to institute’s standards. Radiologic response rates were assessed according to the RECIST v 1.1 guidelines. Histopathological response assessment was conducted locally and reviewed by experienced pathologists (B.A. van de Wiel, R.V. Rawson) according to the guidelines of the INMC, and categorized as being a pathologic complete response (pCR; 0% viable tumor), near-complete response (near-pCR; >0–10% viable tumor), partial response (pPR; >10–50% viable tumor), or non-response (>50% viable tumor; Tetzlaff et al., 2018). All trAEs were reported and graded by the investigators according to the National Cancer Institute Common Terminology Criteria for Adverse Events (CTCAE) v 5.0. Patients were treated until the end of the treatment schedule, unacceptable toxicity, or withdrawal of consent. Discontinuation criteria due to trAEs are described in the study protocol. Quality of life questionnaires were collected at baseline, week 6 (prior to surgery), weeks 12, 24, 36, 48, 60, year 2, and year 3. Tumor biopsies, blood samples, and feces samples for translational analysis were taken at baseline, week 3, week 6, week 12 (blood samples only), and in case of relapse.

#### NanoString RNA gene expression analyses

RNA was isolated from baseline fresh-frozen patients’ tumor biopsies that had sufficient tumor material based on the pathologists’ scoring (at least 30% tumor cells). If the tumor cell percentage was too low, a new biopsy was required. For on-treatment biopsies (week 3), RNA was isolated in case of the presence of tumor bed and/or immune infiltration, independent of the percentage viable tumor. RNA was isolated at the Molecular Diagnostics department of the NKI using the AllPrep DNA/RNA/miRNA Universal Kit (Qiagen; cat: 80224) according to the manufacturer’s protocol. RNA gene expression analysis of baseline (*n* = 44) and on-treatment (*n* = 40) biopsies was conducted using the PanCancer Immune Profiling panel and the NanoString nCounter Analysis System (NanoString Technologies), which captures the read counts of 784 genes. Raw nCounter data were preprocessed through an NKI-developed pipeline for assessment of the IFN-γ signature gene expression. Housekeeping genes and negative control probes were used to normalize the raw expressions and correct for the background noise. The IFN-γ signature gene expression score was calculated from the mean expression of the 10 genes that compose the IFN-γ signature (STAT1, CXCL9, CXCL10, HLA-DRA, GZMA, PRF1, IDO1, CXCL11, CCR5, IFNG) and corrected by an optimization factor based on 49 patients form our OpACIN neo arm B and PRADO reference cohorts. Because the IFN-γ signature was the strongest cytokine gene signature predicting response in our previous cohorts (Blank et al., 2018; Rozeman et al., 2021; Reijers et al., 2022b), we did not compare the IFN-γ signature to other gene signatures in this manuscript. IFN-γ signature gene expression scores were compared using two-tailed unpaired Student’s *t* test for inter-patient comparisons and paired *t* tests for intra-patient comparisons. Since the normalized data were log transformed, the values of NanoString Treg signature (FOXP3) were subtracted from that of CD8 signature (average of CD8A and CD8B) to obtain CD8/Treg ratio. CD8/Treg ratio was compared between baseline and week 3, within each arm, using paired Student’s *t* test.

#### Immunohistochemistry analyses

Immunohistochemistry of the FFPE tumor samples was performed on a BenchMark Ultra autostainer (Ventana Medical Systems). Briefly, paraffin sections were cut at 3 µm, heated at 75°C for 28 min, and deparaffinized in the instrument with EZ prep solution (Ventana Medical Systems). Heat-induced antigen retrieval was carried out using Cell Conditioning 1 (CC1; Ventana Medical Systems) for 48 min at 95°C. PD-L1 clone 22C3 (DAKO) was detected using 1:40 dilution. Bound antibody was visualized using the OptiView DAB Detection Kit (Ventana Medical Systems). Slides were counterstained with hematoxylin II and bluing reagent (Ventana Medical Systems). After staining, slides were scanned with the P1000 (Sysmex). An experienced pathologist determined the tumor proportion score (TPS; the percentage of tumor cells with complete or partial membranous staining at any intensity) using Slide Score (https://www.slidescore.com). The TPS was classified as being <1%, 1–50%, >50%, or not evaluable (due to pigmentation or little to no tumor cells).

#### Study oversight

The trial was conducted in accordance with the protocol and Good Clinical Practice Guidelines as defined by the International Conference on Harmonization and the principles of the Declaration of Helsinki. The protocol was approved by the local ethics committee of the NKI (sponsor). 4SC provided funding for the trial and provided domatinostat tablets. All participating patients provided written informed consent before enrolment.

#### DSMB evaluation

A DSMB was established, consisting of Prof. Dr. D. Schadendorf (University Hospital Essen), Prof. Dr. R. Dummer (University of Zurich), Dr. V. Sondak (Moffitt Cancer Center), and Dr. S. Suciu (European Organization for Research and Treatment of Cancer). The DSMB consultation plan was to evaluate the safety and feasibility of all treatment regimens based on the predefined stopping rules for feasibility after inclusion of 5 and 10 patients in each arm, and to decide on dose (de-)escalation of the triple therapy in arm D. The DSMB advised to continue in arm D with the same domatinostat dose (200 mg QD) after inclusion of 5 patients due to two cases of grade 3 immunotherapy-related toxicity, but advised to increase the dose of domatinostat to 200 mg BID (in arm D-exp) after 10 patients. After inclusion of four patients in arm D-exp, the DSMB agreed that it was reasonable to stop further inclusion of patients due to the dose limiting skin toxicity and lack of improved efficacy.

### Online supplemental material

Fig. S1 shows extended data on the effect of combination domatinostat with anti-PD1 ± anti-CTLA4 on tumor growth in MeVa2.1.dOVA tumor–bearing C57BL/6 mice, the effect of domatinostat on tumor growth in NGS mice, the frequency of CD8^+^ T cells, CD4^+^ FoxP3^+^ Tregs, CD206^+^, and CD206^−^ macrophages in the tumor after 13 d of treatment, and gene set enrichment analysis depicting Hallmark IFN-γ response after treatment. Fig. S2 displays the flow chart of the DONIMI trial. Fig. S3 shows the event-free survival and overall survival per treatment arm. Table S1 summarizes the adverse events of the immunotherapy ± domatinostat, targeted therapy, and surgery. Table S2 shows the radiologic response rates after 6 wk of neoadjuvant therapy.

## Supplementary Material

Table S1 shows all immunotherapy ± domatinostat-related adverse events used in this study.

Table S2 shows radiological response on week 6 CT scan according to RECIST v 1.1 4.

## Data Availability

RNA sequencing data from the preclinical studies are deposited at the Gene Expression Omnibus under accession no. GSE217162. To minimize the risk of patient reidentification, deidentified individual patient-level clinical data are available under restricted access. Upon scientifically sound request, data access can be obtained via the NKI’s scientific repository at repository@nki.nl, which will contact the corresponding author (C.U. Blank). Data requests will be reviewed by the institutional review board of the NKI, and will require the requesting researcher to sign a data access agreement with the NKI.
