# Supplementary material for: IFN-γ signature enables selection of neoadjuvant treatment in patients with stage III melanoma
Source: J Exp Med. 2023 Mar 15;220(5):e20221952. doi: 10.1084/jem.20221952 (PMC10037109; doi:10.1084/jem.20221952)
Supplement: Table S1 — shows all immunotherapy ± domatinostat-related adverse events used in this study. [file JEM_20221952_TableS1.docx]

# Table S1A. All immunotherapy +/- domatinostat-related adverse events

| **Adverse event** | **A: IFN-γ high**  **NIVO**  **(N=10)** | | | | **B: IFN-γ high**  **NIVO + DOM BID**  **(N=10)** | | | | **C: IFN-γ low**  **NIVO + DOM BID**  **(N=10)** | | | | **D: IFN-γ low**  **IPI + NIVO + DOM**  **QD (n=10)** | | | | **D-exp: IFN-γ low**  **IPI + NIVO + DOM**  **BID (n=4)** | | | |
| --- | --- | --- | --- | --- | --- | --- | --- | --- | --- | --- | --- | --- | --- | --- | --- | --- | --- | --- | --- | --- |
|  | **Grade 1-2** | | **Grade 3** | | **Grade 1-2** | | **Grade 3** | | **Grade 1-2** | | **Grade 3** | | **Grade 1-2** | | **Grade 3** | | **Grade 1-2** | | **Grade 3** | |
| **Any adverse event** | **9** | **90%** | **1** | **10%** | **8** | **80%** | **2** | **20%** | **6** | **60%** | **4** | **40%** | **8** | **80%** | **2** | **20%** | **0** | **0%** | **0** | **100%** |
| Fatigue | 7 | 70% | 0 | - | 4 | 40% | 0 | - | 6 | 60% | 0 | - | 6 | 60% | 0 | - | 1 | 25% | 0 | - |
| Skin rash | 4 | 40% | 0 | - | 3 | 30% | 2 | 20% | 3 | 30% | 4 | 40% | 2 | 20% | 0 | - | 1 | 25% | 3 | 75% |
| Pruritus | 5 | 50% | 0 | - | 3 | 30% | 0 | - | 3 | 30% | 0 | - | 4 | 40% | 0 | - | 3 | 75% | 0 | - |
| ALT increased | 2 | 20% | 0 | - | 2 | 20% | 0 | - | 4 | 40% | 1 | 10% | 3 | 30% | 0 | - | 3 | 75% | 0 | - |
| AST increased | 2 | 20% | 0 | - | 2 | 20% | 0 | - | 4 | 40% | 0 | - | 3 | 30% | 0 | - | 2 | 50% | 1 | 25% |
| Headache | 2 | 20% | 0 | - | 3 | 30% | 0 | - | 4 | 40% | 0 | - | 2 | 20% | 0 | - | 2 | 50% | 0 | - |
| Arthralgia | 4 | 40% | 0 | - | 5 | 50% | 0 | - | 0 | - | 0 | - | 3 | 30% | 0 | - | 0 | - | 0 | - |
| Dry mouth | 2 | 20% | 0 | - | 4 | 40% | 0 | - | 1 | 10% | 0 | - | 1 | 10% | 0 | - | 1 | 25% | 0 | - |
| Myalgia | 2 | 20% | 0 | - | 2 | 20% | 0 | - | 1 | 10% | 0 | - | 3 | 30% | 0 | - | 1 | 25% | 0 | - |
| Vitiligo | 5 | 50% | 0 | - | 3 | 30% | 0 | - | 0 | - | 0 | - | 1 | 10% | 0 | - | 0 | - | 0 | - |
| Fever | 0 | - | 0 | - | 1 | 10% | 0 | - | 3 | 30% | 1 | 10% | 1 | 10% | 0 | - | 2 | 50% | 0 | - |
| Lipase increased | 2 | 20% | 0 | - | 3 | 30% | 0 | - | 0 | - | 0 | - | 2 | 20% | 0 | - | 1 | 25% | 0 | - |
| Nausea | 0 | - | 0 | - | 2 | 20% | 0 | - | 2 | 20% | 0 | - | 3 | 30% | 0 | - | 0 | - | 0 | - |
| Diarrhea | 1 | 10% | 0 | - | 0 | - | 0 | - | 0 | - | 0 | - | 4 | 40% | 1 | 10% | 0 | - | 0 | - |
| Hyperthyroidism | 1 | 10% | 0 | - | 0 | - | 0 | - | 1 | 10% | 0 | - | 3 | 30% | 0 | - | 0 | - | 0 | - |
| Infusion related reaction | 1 | 10% | 0 | - | 1s | 10% | 0 | - | 1 | 10% | 0 | - | 2 | 20% | 0 | - | 0 | - | 0 | - |
| Amylase increased | 0 | - | 0 | - | 2 | 20% | 0 | - | 1 | 10% | 0 | - | 2 | 20% | 0 | - | 0 | - | 0 | - |
| Gastrointestinal pain | 1 | 10% | 0 | - | 1 | 10% | 0 | - | 1 | 10% | 0 | - | 1 | 10% | 0 | - | 0 | - | 0 | - |
| Hypothyroidism | 1 | 10% | 0 | - | 0 | - | 0 | - | 1 | 10% | 0 | - | 2 | 20% | 0 | - | 0 | - | 0 | - |
| Platelet count decreased | 0 | - | 0 | - | 0 | - | 0 | - | 1 | 10% | 0 | - | 1 | 10% | 0 | - | 2 | 50% | 0 | - |
| Dysgeusia | 0 | - | 0 | - | 0 | - | 0 | - | 2 | 20% | 0 | - | 1 | 10% | 0 | - | 0 | - | 0 | - |
| Dyspnea | 0 | - | 0 | - | 1 | 10% | 0 | - | 0 | - | 0 | - | 1 | 10% | 0 | - | 1 | 25% | 0 | - |
| GGt increased | 0 | - | 0 | - | 0 | - | 0 | - | 1 | 10% | 0 | - | 1 | 10% | 0 | - | 1 | 25% | 0 | - |
| Oral mucositis | 0 | - | 0 | - | 0 | - | 0 | - | 1 | 10% | 0 | - | 0 | - | 0 | - | 0 | - | 1 | 25% |
| Acute kidney injury | 0 | - | 0 | - | 0 | - | 0 | - | 0 | - | 0 | - | 0 | - | 1 | 10% | 0 | - | 0 | - |
| Colitis | 0 | - | 1 | 10% | 0 | - | 0 | - | 0 | - | 0 | - | 0 | - | 0 | - | 0 | - | 0 | - |
| Pneumonitis | 0 | - | 0 | - | 0 | - | 1 | 10% | 0 | - | 0 | - | 0 | - | 0 | - | 0 | - | 0 | - |
| Data are n (%). Treatment-related adverse events that occurred in at least >5% of patients and all grade 3-4 are displayed in the table. No treatment-related grade 4 or 5 adverse events were observed. *ALT = alanine aminotransferase; AST = aspartate aminotransferase; BID = twice daily; DOM = domatinostat; IFN-γ = interferon gamma; IPI = ipilimumab; NIVO = nivolumab; QD = once daily.* | | | | | | | | | | | | | | | | | | | | |

# Table S1B. Targeted therapy-related adverse events

| **Adverse event** | **A: IFN-γ high**  **NIVO**  **(N=0)** | | | | **B: IFN-γ high**  **NIVO + DOM BID**  **(N=2)** | | | | **C: IFN-γ low**  **NIVO + DOM BID**  **(N=2)** | | | | **D: IFN-γ low**  **IPI + NIVO + DOM**  **QD (n=2)** | | | | **D-exp: IFN-γ low**  **IPI + NIVO + DOM**  **BID (n=3)** | | | |
| --- | --- | --- | --- | --- | --- | --- | --- | --- | --- | --- | --- | --- | --- | --- | --- | --- | --- | --- | --- | --- |
|  | **Grade 1-2** | | **Grade 3** | | **Grade 1-2** | | **Grade 3** | | **Grade 1-2** | | **Grade 3** | | **Grade 1-2** | | **Grade 3** | | **Grade 1-2** | | **Grade 3** | |
| **Any adverse event** | **-** | **-** | **-** | **-** | **2** | **100%** | **0** | **0%** | **1** | **50%** | **1** | **50%** | **0** | **0%** | **2** | **100%** | **1** | **33%** | **2** | **66%** |
| Fever | **-** | **-** | **-** | **-** | 2 | 100% | 0 | - | 2 | 100% | 0 | - | 1 | 50% | 0 | - | 3 | 100% | 0 | - |
| ALT increased | **-** | **-** | **-** | **-** | 0 | - | 0 | - | 0 | - | 0 | - | 1 | 50% | 0 | - | 1 | 33% | 1 | 33% |
| AST increased | **-** | **-** | **-** | **-** | 0 | - | 0 | - | 0 | - | 0 | - | 1 | 50% | 0 | - | 1 | 33% | 1 | 33% |
| Fatigue | **-** | **-** | **-** | **-** | 1 | 50% | 0 | - | 1 | 50% | 0 | - | 0 | - | 0 | - | 1 | 33% | 0 | - |
| Skin rash | **-** | **-** | **-** | **-** | 0 | - | 0 | - | 1 | 50% | 0 | - | 1 | 50% | 0 | - | 1 | 33% | 0 | - |
| Creatinine increase | **-** | **-** | **-** | **-** | 0 | - | 0 | - | 0 | - | 0 | - | 0 | - | 0 | - | 0 | - | 1 | 33% |
| Data are n (%). Targeted therapy-related adverse events that occurred in at least 3 patients and all grade 3 are displayed in the table. *BID = twice daily; DOM = domatinostat; IFN-γ = interferon gamma; IPI = ipilimumab; NIVO = nivolumab; QD = once daily.* Table S1C. Surgery-related adverse events | | | | | | | | | | | | | | | | | | | | |
|  | | | | | | | | | | | | | | | | | | | | |

| **Adverse event** | **A: IFN-γ high**  **NIVO**  **(N=10)** | | | | **B: IFN-γ high**  **NIVO + DOM BID**  **(N=10)** | | | | **C: IFN-γ low**  **NIVO + DOM BID**  **(N=10)** | | | | **D: IFN-γ low**  **IPI + NIVO + DOM**  **QD (n=10)** | | | | **D-exp: IFN-γ low**  **IPI + NIVO + DOM**  **BID (n=4)** | | | |
| --- | --- | --- | --- | --- | --- | --- | --- | --- | --- | --- | --- | --- | --- | --- | --- | --- | --- | --- | --- | --- |
|  | **Grade 1-2** | | **Grade 3** | | **Grade 1-2** | | **Grade 3** | | **Grade 1-2** | | **Grade 3** | | **Grade 1-2** | | **Grade 3** | | **Grade 1-2** | | **Grade 3** | |
| **Any adverse event** | **7** | **70%** | **3** | **30%** | **9** | **90%** | **1** | **10%** | **6** | **60%** | **1** | **10%** | **8** | **80%** | **2** | **20%** | **2** | **50%** | **1** | **25%** |
| Seroma | 8 | 80% | 0 | - | 7 | 70% | 1 | 10% | 5 | 50% | 1 | 10% | 7 | 70% | 0 | - | 2 | 50% | 0 | - |
| Wound infection | 1 | 10% | 3 | 30% | 4 | 40% | 1 | 10% | 3 | 30% | 0 | - | 4 | 40% | 2 | 20% | 0 | - | 1 | 25% |
| Wound dehisence | 1 | 10% | 0 | - | 3 | 30% | 0 | - | 1 | 10% | 0 | - | 4 | 40% | 0 | - | 1 | 25% | 0 | - |
| Edema limbs | 1 | 10% | 0 | - | 3 | 30% | 0 | - | 1 | 10% | 0 | - | 3 | 30% | 0 | - | 0 | - | 0 | - |
| Joint range of motion decreased | 0 | - | 0 | - | 2 | 20% | 0 | - | 2 | 20% | 0 | - | 1 | 10% | 0 | - | 0 | - | 0 | - |
| Paresthesia | 1 | 10% | 0 | - | 3 | 30% | 0 | - | 0 | - | 0 | - | 1 | 10% | 0 | - | 0 | - | 0 | - |
| Lymphedema | 2 | 20% | 0 | - | 1 | 10% | 0 | - | 1 | 10% | 0 | - | 0 | - | 0 | - | 0 | - | 0 | - |
| Post-operative pain | 2 | 20% | 0 | - | 1 | 10% | 0 | - | 0 | - | 0 | - | 0 | - | 0 | - | 0 | - | 0 | - |
| Data are n (%). Surgery-related adverse events that occurred in at least >5% of patients and all grade 3 are displayed in the table. No grade 4 or 5 surgery-related adverse events were observed. *BID = twice daily; DOM = domatinostat; IFN-γ = interferon gamma; IPI = ipilimumab; NIVO = nivolumab; QD = once daily.* | | | | | | | | | | | | | | | | | | | | |
