# Supplementary material for: IFN-γ signature enables selection of neoadjuvant treatment in patients with stage III melanoma
Source: J Exp Med. 2023 Mar 15;220(5):e20221952. doi: 10.1084/jem.20221952 (PMC10037109; doi:10.1084/jem.20221952)
Supplement: Table S2 — shows radiological response on week 6 CT scan according to RECIST v 1.1 4. [file JEM_20221952_TableS2.docx]

# Table S2. Radiological response on week 6 CT-scan according to RECISTv1.1

| **Radiological response** | **A: IFN-γ high**  **NIVO**  **(N=10)** | | **B: IFN-γ high**  **NIVO + DOM BID** **(N=10)** | | **C: IFN-γ low**  **NIVO + DOM**  **BID (N=10)** | | **D: IFN-γ low**  **IPI+ NIVO+ DOM**  **QD (N=10)** | | **D-exp: IFN-y low**  **IPI+ NIVO+ DOM**  **BID (N=4)** | |
| --- | --- | --- | --- | --- | --- | --- | --- | --- | --- | --- |
| **ORR (%, 95% CI)** | **5** | **(50%, 19-81%)** | **7** | **(70%, 35-93%)** | **0** | **(0%, 0-31%)** | **4** | **(40%, 12-74%)** | **0** | **(0%, 0-60%)** |
| CR | 1 | (10%) | 3 | (30%) | - | - | 1 | (10%) | - | - |
| PR | 4 | (40%) | 4 | (40%) | - | - | 3 | (30%) | - | - |
| SD | 5 | (50%) | 3 | (30%) | 9 | (90%) | 1 | (10%) | 3 | (75%) |
| PD  Local progression  Distant metastases | - | - | - | - | - | - | 5  3  2 | (50%)  (30%)  (20%) | 1  1  0 | (25%)  (25%)  (0%) |
| Not evaluable | - | - | - | - | 1* | (10%) | - | - | - | - |
| ** The CT-scan of this patient was not evaluable because surgery was brought forward due to impending irresectability*  *BID = twice daily; CR = complete response; DOM = domatinostat; IFN-γ = interferon gamma; IPI = ipilimumab; NIVO = nivolumab; QD = once daily; ORR = objective response rate; PD = progressive disease; PR = partial response.* | | | | | | | | | | |
